# Comparison of transcriptional profiles of *Clostridium thermocellum* grown on cellobiose and pretreated yellow poplar using RNA-Seq

**DOI:** 10.3389/fmicb.2014.00142

**Published:** 2014-04-11

**Authors:** Hui Wei, Yan Fu, Lauren Magnusson, John O. Baker, Pin-Ching Maness, Qi Xu, Shihui Yang, Andrew Bowersox, Igor Bogorad, Wei Wang, Melvin P. Tucker, Michael E. Himmel, Shi-You Ding

**Affiliations:** ^1^Biosciences Center, National Renewable Energy LaboratoryGolden, CO, USA; ^2^Center for Plant Genomics, Iowa State UniversityAmes, IA, USA; ^3^National Bioenergy Center, National Renewable Energy LaboratoryGolden, CO, USA

**Keywords:** *Clostridium thermocellum*, transcriptomics, RNA-Seq, pretreated yellow poplar (PYP), cellobiose, cellulosome, ethanol, hydrogen

## Abstract

The anaerobic, thermophilic bacterium, *Clostridium thermocellum*, secretes multi-protein enzyme complexes, termed cellulosomes, which synergistically interact with the microbial cell surface and efficiently disassemble plant cell wall biomass. *C. thermocellum* has also been considered a potential consolidated bioprocessing (CBP) organism due to its ability to produce the biofuel products, hydrogen, and ethanol. We found that *C. thermocellum* fermentation of pretreated yellow poplar (PYP) produced 30 and 39% of ethanol and hydrogen product concentrations, respectively, compared to fermentation of cellobiose. RNA-seq was used to analyze the transcriptional profiles of these cells. The PYP-grown cells taken for analysis at the late stationary phase showed 1211 genes up-regulated and 314 down-regulated by more than two-fold compared to the cellobiose-grown cells. These affected genes cover a broad spectrum of specific functional categories. The transcriptional analysis was further validated by sub-proteomics data taken from the literature; as well as by quantitative reverse transcription-PCR (qRT-PCR) analyses of selected genes. Specifically, 47 cellulosomal protein-encoding genes, genes for 4 pairs of SigI-RsgI for polysaccharide sensing, 7 cellodextrin ABC transporter genes, and a set of NAD(P)H hydogenase and alcohol dehydrogenase genes were up-regulated for cells growing on PYP compared to cellobiose. These genes could be potential candidates for future studies aimed at gaining insight into the regulatory mechanism of this organism as well as for improvement of *C. thermocellum* in its role as a CBP organism.

## Introduction

Microbial conversion of biomass to biofuels is an attractive route for biofuel development, but an essential challenge is to increase the microbial capacity both for overcoming the biomass recalcitrance and for converting the biomass-derived sugars to biofuels (Himmel et al., 2007; Alper and Stephanopoulos, 2009). *Clostridium thermocellum*, a gram-positive, thermophilic, anaerobic bacterium, is one of the model consolidated bioprocessing (CBP) systems used to study the enzymatic hydrolysis of cellulosic biomass to produce fuels (Islam et al., 2006; Brown et al., 2012; Yang et al., 2012). The following features of *C. thermocellum* contribute to its suitability as a model cellulolytic, biofuel-producing bacterium: (1) It produces cellulosomes, a type of highly organized multi-protein enzyme complexes, which have been shown to be highly efficient enzyme systems in deconstructing plant cell wall, especially in degrading the recalcitrant substrate crystalline cellulose (Bayer et al., 1998, 2004). (2) It carries out mixed-product fermentation, producing ethanol, H_2_ and numerous organic acids including acetate, formate, and lactate (Demain et al., 2005; Islam et al., 2006). (3) It is suitable for both submerged and solid-state fermentation (Bayer et al., 2007), the latter having similarity to compost in the set-up of feedstock (Wei et al., 2012). (4) Its genome sequence is available (http://genome.jgi-psf.org/cloth/cloth.info.html) and many of the cellulolytic enzymes are identified and biochemically characterized. The knowledge gained from studies of this species will benefit work on other clostridial species of industrial interest, such as *C. acetobutylicum*, known to produce the potential fuels acetone, ethanol, and butanol (Cooksley et al., 2012).

Fulfilling this potential will require a more in-depth understanding of the metabolic and genetic mechanisms by which *C. thermocellum* utilizes recalcitrant biomass substrate. So far, a number of studies have been carried out on *C. thermocellum*, such as transcriptomic analysis of stress responses to ethanol, furfural, and heat during growth on pure sugars (i.e., cellobiose) (Yang et al., 2012), time course studies of cell growth on pure crystalline cellulose (Avicel) (Raman et al., 2011), and comparisons of growth on pure crystalline cellulose (Sigmacel 50) and cellobiose (Riederer et al., 2011). However, despite reports on the sub-proteomic analyses of cellulosomes from *C. thermocellum* grown on Avicel (Gold and Martin, 2007) and on combinations of Avicel with pectin and/or xylan, or on pretreated switchgrass (Raman et al., 2009), there were no genome-wide transcriptomic studies reported for growth *on* pretreated woody plant biomass until most recently a transcriptomic analysis comparing *C. thermocellum* cells grown on pretreated switchgrass and woody biomass, cottonwood (*Populus trichocarpa × Populus deltoids*; black cottonwood × eastern cottonwood in common names), was *published on* December 2, 2013 (Wilson et al., 2013a), after the submission of this manuscript.

Woody biomass has been found to be more recalcitrant to enzyme digestion than is herbaceous biomass. For example, whereas the Recalcitrance Index for switchgrass is ~0.35, the index for hardwoods, such as yellow poplar, is 0.56, indicating that hardwoods are more difficult to be degraded (Wei et al., 2009). *Previous* studies have shown that cellulolytic bacteria grown on different lignocellulosic substrates have different levels of glycoside hydrolase (GH) activities (Irwin et al., 2003). As such C. *thermocellum* grown on pretreated woody plant biomass is likely to have distinctive responsive genes, as well as different composition of cellulolytic enzymes different from those grown on herbaceous biomass. Recently, we found that species of the genus *Clostridium* (including *C. thermocellum*) were among the dominant species, comprising 6.3% of the total, in an anaerobic community decomposing yellow poplar wood chips (van der Lelie et al., 2012). In parallel, we studied the yellow poplar compost system (Wei et al., 2012), in which *Clostridium* was also found to be one of the dominant bacteria (data not shown). These results prompted us to explore *C. thermocellum* grown on dilute acid-pretreated yellow poplar (PYP) as a single-species model for the plant cell wall-degradation. PYP is a widely-utilized feedstock in process-development for conversion of biomass to fuels and chemicals, and it is important to identify specific enzymes that this organism calls into action to attack this form of the feedstock/substrate.

PYP has been previously demonstrated that 60% conversion to simple sugars can be achieved with a loading of ~8.4 mg per g biomass cellulose of a mixture of *Trichoderma reesei* CBHI and *Acidothermus cellulolyticus* E1 (95%: 5% on molar basis) (Vinzant et al., 1994; Baker et al., 1997). In this study, *C. thermocellum* was bench fermented using PYP and cellobiose as sole carbon sources, respectively, and transcriptional profiles were analyzed. RNA-seq is a recently developed technology for transcriptome profiling, which uses next-generation sequencing to reveal the presence and quantity of RNA in biological samples. The goals of this study are two-fold: first, we identified genes responsive for degradation of recalcitrant biomass substrates in PYP- vs. cellobiose-grown *C. thermocellum* cells. Secondly, we specifically focused on candidate genes related to cellulosome, cellodextrin transport, polysaccharide signal transduction and end-product synthesis related genes. These candidate genes are likely to be valuable for mechanism study; as well as for protein-engineering to further improve the abilities of this already potent organism to degrade the cell walls of recalcitrant biomass feedstocks.

## Materials and methods

**Figure 1:**
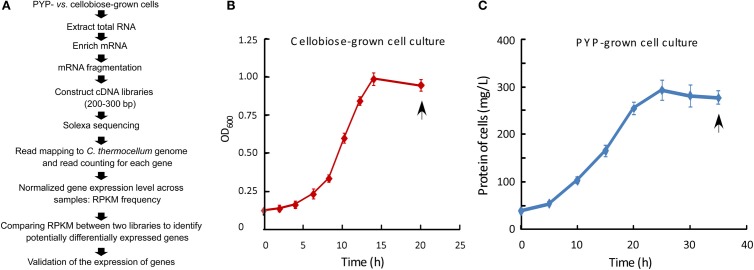
**Flowchart for experimental procedures, along with growth curves for cellobiose- and PYP-grown *C. thermocellum* cells. (A)** Flowchart outlining the experimental procedures; RPKM, reads per kilobase of exon model per million mapped reads. **(B)** OD_600_ for cellobiose-grown cells was measured by spectrophotometry at 600 nm (OD_600_). **(C)** Cell pellet protein concentration for pretreated yellow poplar (PYP)-grown cell culture. The doubling time for cellobiose- and PYP-grown cells was 3.3 and 7.8 h, respectively. Data are presented as the mean (±SE) of 3 replicates. Arrows indicate the time points sampled for transcriptomic analyses.

Figure 1A shows the overall experimental approach we designed to investigate the *C. thermocellum* utilization of pretreated biomass substrates as reflected by changes in the cell's transcriptome. Details of the experimental approach are described in the following sections.

### Carbon and lignocellulosic substrates

Cellobiose and other chemical compounds were purchased from Sigma (St. Louis, MO). PYP was prepared as described previously (Tucker et al., 1998). Briefly, the milled yellow biomass (20% solids loading) was pretreated in 0.21% w/w H_2_SO_4_ at 200°C for 4 min. The resultant PYP contained ~65% cellulose, 4% xylan, and 31% lignin (dry weight basis). PYP was exhaustively washed with deionized water until the pH reached that of deionized water, prior to being used in medium preparation as described below.

### Microorganisms and culture conditions

*C. thermocellum* ATCC 27405 was routinely cultured at 60°C anaerobically in 30-mL serum bottles containing 10 mL of ATCC medium 1191 containing cellobiose, and was subcultured with 2% inoculum taken at exponential **g**rowth phase. For this study, similarly to practices in the literature (Gold and Martin, 2007; Rydzak et al., 2009), 100-mL batch culture in 250-mL serum bottles was used for the growth of the strain anaerobically at 58°C in ATCC medium 1191, containing 0.30% (w/v) cellobiose as the control carbohydrate substrate, or 0.44% PYP as biomass substrate (sugar-content equivalent to 0.30% cellobiose) with an agitation of 130 rpm. Cell growth was monitored based on either the measurement of optical density (OD) by spectrophotometry at 600 nm (OD_600_) for cellobiose-grown culture, or the increase in pellet protein amount for PYP-grown culture using the method described in literature (Raman et al., 2009). Three independent sets of PYP- vs. cellobiose-grown cell cultures, harvested at the late stationary phase (20 h for cellobiose-grown cells and 36 h for PYP-grown cells), were carried through the downstream RNA extraction preparations. Two sets of RNA preparations were used for cDNA library construction and RNA-Seq, whereas all three sets of RNA preparations were used for qRT-PCR analysis in verifying RNA-Seq data of selected genes.

### Measurement of H_2_ and ethanol, and isolation of bacterial total RNA

Cells were harvested for RNA isolation in late stationary phase after the hydrogen concentration at the headspace was analyzed using GC method, wherein an Agilent 7890A GC equipped with a Supelco 60/80 molsieve 5A column was used for the measurement of H_2_. The cell culture was centrifuged at high speed (8000 × g) for 5 min to pellet the bacterial cells. Cell-free culture supernatants were used to measure ethanol concentration by using HPLC (Agilent) with a refractive index detector (Shimadzu, Kyoto, Japan). All samples were filtered through a 0.45-μm filter before HPLC analysis. The organic acids were separated in an Aminex HPX-87H column (Bio-Rad) running at a flow rate of 0.6 ml/min at 50°C, with 4 mM H_2_SO_4_ as eluent.

The cell pellets collected from 10 mL culture were used for RNA extraction, using a combined protocol (Yang et al., 2012), in which the TRIzol (Invitrogen, Carlsbad, CA) extraction aqueous phase was mixed with an equal volume of 70% ethanol, and then applied to the column of Qiagen RNeasy Mini Kit (Qiagen, Valencia, CA) for purification according to the manufacturer's instructions to obtain total RNAs. 7.5 μg total RNAs were mixed with 3 μL DNase I (Invitrogen; 1 U/μL) in 3 μL 10x DNase I reaction buffer, with adding RNase-free water added up to a total volume of 30 μL, and incubated at room temperature for 15 min. The DNase I was inactivated by adding 3 μL of 25 mM EDTA and heating for 10 min at 65°C. The DNase I treated total RNA was precipitated by the ethanol-glycogen method and re-dissolved in 15 μL of 1 mM EDTA. Since the total RNAs contains 75% rRNA (Chen and Duan, 2011), mRNA was enriched by using the MICROBExpress Bacterial mRNA Enrichment Kit (cat # AM1905, Ambion, Life Technologies, Austin, TX) to remove 16 and 23 s rRNAs. The resultant RNAs were quantified and analyzed for integrity on the Agilent 2100 Bioanalyzer, and used for cDNA library construction as described below.

### cDNA library construction, sequencing, and read mapping

cDNA libraries were constructed with the RNA-Seq sample preparation kit (RS-100-0801, Illumina, San Diego, CA), using the procedure provided by the manufacturer. Briefly, the above enriched mRNA samples were fragmented, and then annealed to random hexamers and reverse transcribed. After second strand synthesis, end repair, and A-tailing, cDNA fragment ends were ligated to adapters that were complementary to sequencing primers. Resultant cDNA libraries were size separated on agarose gels with ~200 bp fragments being excised, and amplified by 15 cycles of PCR. The prepared cDNA libraries were sequenced on an Illumina Genome Analyzer II by the Michigan State University DNA facility, using the standard protocols and running for 75 cycles of data acquisition. Solexa reads were aligned to the reference genome sequence of *C. thermocellum* strain (ATCC 27405) deposited at NCBI (Accession: NC_009012.1) using Bowtie (Langmead et al., 2009). The reference genome is 3.8-Mb long with 3305 predicted genes, including 71 structural RNA genes, according to the NCBI website for the genome (http://www.ncbi.nlm.nih.gov/genome/?term=Clostridium+thermocellum) accessed on April 28, 2010. The best end-to-end alignment (ties broken by read quality) was used with no more than two mismatches. The reads that cannot be unambiguously mapped were not included for further analysis. The above alignment approach and criteria were similar to those used in literature (Jia et al., 2009; Lin et al., 2011).

### Gene expression normalization and identification of differentially expressed genes

After read mapping, the midpoint of the read-reference alignment was used to determine which gene that read belongs to (or was derived from), and the RNA-seq read counts for each gene can be then calculated. To facilitate comparison of gene expression levels, both within and between two samples, we quantified and normalized gene expression levels by calculating the reads per kilobase of exon model per million mapped reads (RPKM), i.e., calculating the number of reads mapped per kilobase of exon model divided by the total number of mapped reads in the whole dataset (Mortazavi et al., 2008). As an indicator of gene expression level, the RPKM is widely recognized as a measure of Solexa read density that reflects the molar concentration of a transcript in the starting RNA sample, thus making the normalized gene expression levels comparable within and among samples.

The fold-change for the expression of an individual gene was calculated by taking the ratio of RPKM in PYP-grown cells to that in cellobiose-grown cells (RPKM_PYP_/RPKM_cellobiose_). The cutoff value for defining a gene as “differentially expressed” is either a two-fold increase (with the value of RPKM_PYP_/RPKM_cellobiose_ larger than 2.0), or a two-fold decrease (with the value of RPKM_PYP_/RPKM_cellobiose_ less than 0.5).

### Statistical analysis

The log2-transformed raw RPKM dataset was imported into the statistical analysis software JMP Genomics 6.0 software (SAS Institute, NC), and the data of PYP- and cellobiose-grown cell samples were normalized together using the LOWESS normalization algorithm within JMP Genomics. To determine if the differential expression levels between PYP- and cellobiose-grown cell samples were statistically significant, the normalized Log2(RPKM) data were subjected to One-Way analysis of variance (ANOVA) as described in literature (Yang et al., 2012, 2013; Wilson et al., 2013b). The False Discovery Rate (FDR) testing method was used with a significance threshold of *p* < 0.05 being considered statistically significant.

### Biological interpretation of differentially expressed genes

The identified, differentially expressed genes were interpreted and discussed in the context of biological processes and functions, using clusters of orthologous groups (COG) and Carbohydrate-Active enZYmes (CAZy) analyses of proteins (www.cazy.org/).

### Quantitative reverse transcription-PCR (qRT-PCR)

Based on their potential functional importance, 12 genes were selected for validating the results of the RNA-Seq analysis. The primers for these genes were either based on literature or designed with Primer Express 3.0 software (Applied Biosystems), and are described along with the PCR results in the Results and Discussion section. Total RNA was extracted from three sets of independent cultures grown on PYP vs. cellobiose as described above, and then converted to cDNA by random priming, using the SuperScript II kit (Invitrogen, San Diego, CA). PCR reactions were run in triplicate using procedure as previously described (Wei et al., 2012). The transcription level of genes was determined according to the 2^−ΔΔCT^ method, using RecA as a reference gene for the normalization of gene expression levels (Stevenson and Weimer, 2005).

### Correlation analysis for cellulosome-related gene expression and protein abundance

Two sets of quantitative cellulosomal protein data of cellobiose-grown *C. thermocellum* cells at late stationery phase were retrieved from literature (Gold and Martin, 2007; Raman et al., 2009), and plotted against our sub-dataset of log2(RPKM) values of cellulosomal genes in cells grown on the same substrate at the same culture phase in this study. Pearson correlation coefficient values were calculated using Microsoft Excel (Microsoft Corporation, Redmond, WA, USA), and used as an indicator for the degree of correlation for the compared pairs.

### Compositional analysis of pretreated yellow poplar residues

The PYP residues from PYP-grown *C. thermocellum* culture were collected by centrifugation at low speed (100 × g) for 2 min to precipitate the insoluble substrate not consumed by the bacterial cells in the culture. Such centrifugation speed has been used in the literature to remove any insoluble substrate in *C. thermocellum* culture (Dror et al., 2005). Compositional analysis of the collected PYP residue was performed by the National Bioenergy Center, National Renewable Energy Laboratory, using method described in literature (Templeton et al., 2010).

## Results and discussion

### *C. thermocellum* growth and the production of hydrogen and ethanol

The first step of this study, as illustrated in Figures 1A–C, is the growth of *C. thermocellum* ATCC 27405 with two types of carbohydrate substrates, PYP being compared with cellobiose. GC analysis of gas composition in the headspace of batch cultures at the late stationary phase revealed a production yield of 1.22 vs. 0.92 mole H_2_/mole glucose consumed in cellobiose- and PYP-grown *C. thermocellum*, respectively (Table 1). Furthermore, HPLC analysis of metabolite production in supernatants of harvested cell culture revealed a production yield of 0.51 vs. 0.30 mole ethanol /mole glucose equivalent consumed in cellobiose- and PYP-grown *C. thermocellum*, respectively. Overall, the production of hydrogen and ethanol by PYP-grown *C. thermocellum* are 75 and 58% of those by cellobiose-grown *C. thermocellum*, respectively (Table 1). Wet chemistry analysis of spent PYP solids revealed that 52% of the glucan in the starting PYP substrate was consumed during the fermentations, which can partially explain the lower absolute H_2_ and ethanol productions compared to the culture on cellobiose substrate (in which nearly all cellobiose was depleted at late stationary phase).

**Table 1:** **Hydrogen, ethanol and acetate production at the late stationary phase of *C. thermocellum* culture**.

**Cell culture**	**Yields**			**Acetate/Ethanol**
	**Hydrogen**	**Ethanol**	**Acetate**	
	**(Mole product/Mole glucose equivalent consumed)**			
Cellobiose-grown cells	1.22 ± 0.11	0.51 ± 0.05	0.55 ± 0.07	1.08
PYP-grown cells	0.92 ± 0.09	0.30 ± 0.04	0.27 ± 0.04	0.92
Yield in PYP-grown cells/yield in cellobiose-grown cells (%)	75	58	49	

*The time point for measurements was 20 and 36 h for cellobiose- and PYP-grown cells, respectively. Values for the yields of hydrogen, ethanol and acetate are means ± s.e.m. of three independent experiments. PYP, acid-pretreated yellow poplar*.

### RNA-Seq results

**Figure 2:**
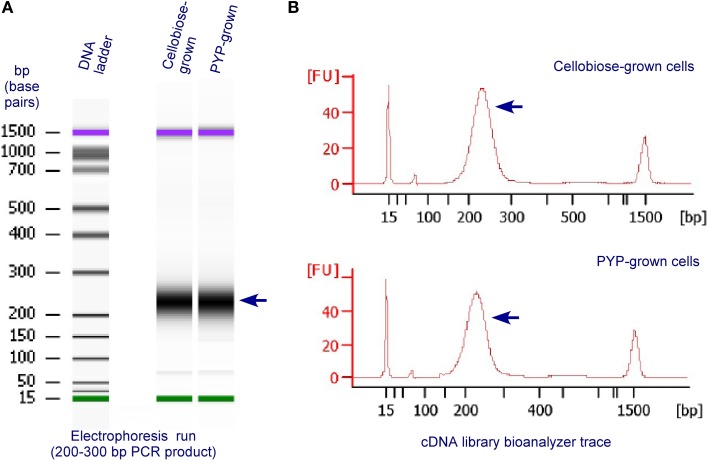
**Validation and quantification of representative, final RNA-Seq cDNA libraries prior to GA II, using an Agilent 2100 Bioanalyzer and the DNA 1000 Nano Chip Kit (Agilent, Technologies, Santa Clara, CA, USA)**. The RNA-Seq cDNA libraries were prepared from RNA extracted from *C. thermocellum* cells grown on cellobiose or PYP. **(A)** Electrophoresis run of the cDNA libraries by the Bioanalyzer; **(B)** bioanalyzer trace of cDNA libraries.

The quality of RNA-Seq cDNA libraries was assessed on a Bioanalyzer prior to GA II (Figure 2). The results showed the size distribution of cDNA library was between 200 and 300 bp, which met the requirement for Solexa sequencing.

For the two PYP-grown cell cDNA library samples, Solexa sequencing from the first cDNA library generated 20.8 million total raw reads, with 14.5 million passing filter reads (70% PF reads); from the second cDNA library generated 19.6 million total raw reads, with 14.1 million passing filter reads (72% PF reads). Out of these PYP-grown cell derived reads, 2.3 and 2.1 millions reads were unambiguously mapped to *C. thermocellum* genome with the criteria set in the Materials and Methods section, respectively. For the two cellobiose-grown cell cDNA library samples, Solexa sequencing from their first cDNA library generated 21.1 million total raw reads, with 15.7 million passing filter reads (74% PF reads); from the second cDNA library generated 19.7 million total raw reads, with 14.0 million passing filter reads (71% PF reads). Out of these cellobiose-grown cell-derived reads, 2.6 and 2.2 millions reads were unambiguously mapped to *C. thermocellum* genome, respectively. The RPKM value for each gene in each condition was calculated as RPKM = Reads number mapped to gene/Length of the gene (kb)/Total reads number (million reads), as described in the Materials and Methods section.

Overall, sequence analysis successfully aligned the Solexa reads to 3081 protein-coding genes and 61 structural RNA genes in cellobiose- and/or PYP-grown *C. thermocellum* mRNA samples, accounting for 95% [i.e., (3081 + 61)/3305] of all *C. thermocellum* genes in the genome, indicating that RNA-Seq analysis in this study achieved comprehensive coverage of the *C. thermocellum* transcriptome. In addition, the above result is consistent with the reports that in the genomes of other microorganisms such as *Saccharomyces cerevisiae* and *S. pombe*, more than 90% of the genes are transcriptionally active and expressed (Nagalakshmi et al., 2008; Wilhelm et al., 2008; Wang et al., 2009). The detailed lists of genes for the RNA-Seq sequences identified in the cellobiose- and PYP-grown cells are presented in Supplementary Data Sheet 1. Each of the RPKM values of cellobiose-grown cells and RPKM of PYP-grown cells was the average of two replicates.

Our data from Solexa-read transcriptome measurement of PYP- vs. cellobiose-grown *C. thermocellum* illustrate some key characteristics of the results. First, the obtained RPKM values for most of the transcripts of the active protein-coding genes in PYP- and cellobiose-grown *C. thermocellum*, are in the range of 1–50,000 (which can be revealed by re-sorting the Supplementary Data Sheet 1). Such range of RPKM values is comparable to the reported range of RPKM values in RNA-Seq whole-transcriptome analysis of other organism samples (Tang et al., 2009).

**Figure 3:**
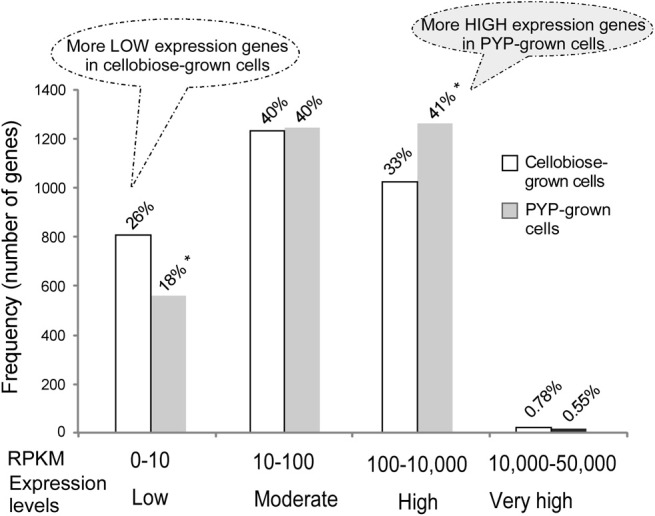
**RPKM frequency histogram of transcripts from RNA-Seq of pretreated yellow poplar (PYP)- vs. cellobiose-grown *C. thermocellum* cells**. The diagram shows the distribution of the number of genes expressed at different RPKM levels. The total number of protein coding genes aligned with the RNA-Seq was 3081; the percentage value above each bar indicates the genes at specific expression level accounting for the proportion of total number of genes. The ^*^ mark indicates that significantly different frequencies (i.e., numbers of genes) were observed between the two RNA-Seq data sets from PYP- vs. cellobiose-grown *C. thermocellum* cells.

The gene expression levels can be classified into four levels: low, moderate, high, and very high, as illustrated in the X axis in Figure 3. While the RNA-Seq data sets of PYP- vs. cellobiose-grown cells had comparable numbers of genes that fall in the moderate expression levels (i.e., both 40% of the active protein-encoding genes), the PYP-grown cells had more highly expressed genes (41 vs. 33% in that of cellobiose-grown culture; Figure 3). In contrast, cellobiose-grown cells had more lowly expressed genes (26 vs. 18% in that PYP-grown culture).

### Changes in global gene expression and clusters of orthologous groups (COG) analysis

To compare the differential expression of genes between PYP- and control cellobiose-grown cells, fold changes (FC) were computed as the ratio of the RPKM values obtained for individual genes in PYP- against cellobiose-grown cells. Analysis of changes in global gene expression had identified 1211 genes that show a two-fold or greater increase in expression (i.e., *FC* ≥ 2.0) or detected in PYP-grown cells only (referred as PYPO genes), and 314 genes with a two-fold or greater decrease (i.e., *FC* ≤ 0.5) in transcript abundance for PYP- against cellobiose-grown cells or detected in cellobiose-grown cells only (referred as CBO genes), as listed in Supplementary Data Sheet 2 with related statistic analyses showing that they were statistically significant.

**Figure 4:**
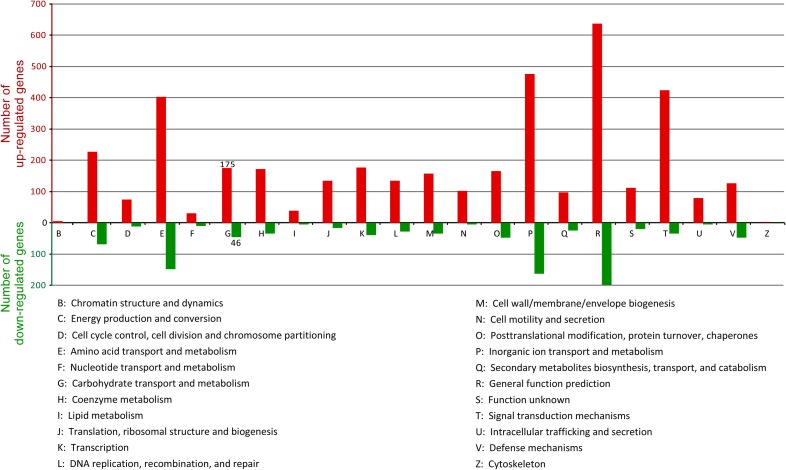
**COG functional categories of the 1211 up- and 314 down-regulated genes in PYP-grown cells compared with cellobiose-grown cells**. Note that since COG annotation classes overlap, the sum of COG annotated genes is larger than the number of total up- and down-regulated genes analyzed.

The COG distribution for these up- and down-regulated genes in the transcriptome was determined and the result is shown in Figure 4. The top two categories for up- and down-regulated genes belong to the categories “general function prediction, [R]” (equivalent to unclassified) and “inorganic ion transport and metabolism, [P].” A closer examination of the distribution chart reveals that for the up-regulated genes, the categories “signal transduction mechanisms, [T],” “amino acid transport and metabolism, [E],” and “energy production and conversion, [C]” (which is important for the energy-consuming process for biofuel end-product production), were also the most well-represented categories in the transcriptome, with the number of induced genes above 200 for each category (Figure 4).

An important category of COG for the degradation and utilization of lignocellulosic substrate is “carbohydrate transport and metabolism, [G],” which includes primarily cellulosome-related genes. In this category, the number of up- and down-regulated genes is 175 and 46, respectively (Figure 4). These differentially expressed genes are described in detail in later section.

### Validation of RNA-Seq results with quantitative reverse transcription-PCR

Quantitative reverse transcription-PCR (qRT-PCR) is a well accepted method for verifying microarray (Ferreira et al., 2010; Yang et al., 2012) and RNA-Seq data (Cusick et al., 2012; Huang et al., 2012; Ji et al., 2013). We used this method to validate the expression patterns of 12 selected genes in independent biological replicates. The selected genes mainly represented different functional categories involving in cellulosome, hydrogen and ethanol production and had a range of FC values based on RNA-Seq. In addition to these 12 genes, the gene RecA was chosen as a reference gene to normalize the real time RT-PCR data because it has been commonly used as reference gene for *C. thermocellum* (Stevenson and Weimer, 2005). As a further confirmation for the use of this gene as reference gene, RecA did not differ in expression in our cell samples grown on two substrates, the values being RPKM 1166 for PYP-grown cells vs. RPKM 1237 for cellobiose-grown cells. The primers for all 13 genes, along with one-by-one comparisons of the fold-changes in expression of each gene as measured by RNA-Seq and qRT-PCR, are listed in Supplementary Data Sheet 3, section 1. Most of the qRT-PCR data matched the RNA-Seq based FC values with a correlation coefficient of 0.95 for the set of 12 selected genes, which indicated that our RNA-Seq result is accurate and the conclusion from RNA-Seq should be reliable.

### Transcriptional changes of cellulosomal components

Cellulosome is an extracellular supramolecular machine that can efficiently degrade crystalline cellulosic substrates and associated plant cell wall materials. We are especially interested in the genes encoding cellulosomal component proteins in response to cellulosic substrates, which can lead to the identification of regulatory or rate-limiting components regarding cellulolysis.

The number of reported cellulosomal genes in the genome of ATCC 27405 strain has been increasing from the initial numbers of 71 (Zverlov et al., 2005) and 72 (Zverlov and Schwarz, 2006), to more recently, 81 genes (Raman et al., 2011). Note that the latter number is consistent with the updated genome annotation. Of these 81 genes, all were detected transcriptionally in this study. The list of 81 cellulosomal genes with their FC values is described in Table 2.

**Table 2:** **List of 81 cellulosomal genes with their RPKM and fold change (FC) values determined in PYP- vs. cellobiose-grown *C. thermocellum* cells**.

**Row no**.	**Gene ID**	**Protein**	**GH, CBM family**	**RPKM of PYP cells**	**RPKM of cellobiose cells**	***FC***
**47 UP-REGULATED GENES (FC ≥ 2.0); ALL STATISTICALLY SIGNIFICANT (P < 0.05)**						
1	Cthe_0798	Acetyl xylan esterase	CE3	483	20	24.3
2	Cthe_0211	LicB	GH16	1624	86	18.8
3	Cthe_0912	XynY	GH10, CE1, CBM22	261	19	13.8
4	Cthe_1890	Dockerin type I cellulosome prot	n/a	45	3	14.0
5	Cthe_1472	CelH	GH5, GH26, CBM11	334	26	12.7
6	Cthe_3080	Cellulosome anchoring prot, cohesin region	n/a	1490	142	10.5
7	Cthe_0412	CelK	GH9, CBM4	1618	202	8.0
8	Cthe_0413	CbhA	GH9, CBM4	367	49	7.5
9	Cthe_0660	β-1,3-glucanase	GH81	4	1	6.7
10	Cthe_3077	CipA	CBM3	2135	363	5.9
11	Cthe_3078	OlpB	n/a	333	59	5.6
12	Cthe_0239	Cellulosome prot, dockerin type I	n/a	50	9	5.4
13	Cthe_2872	CelG	GH5	1038	197	5.3
14	Cthe_0825	CelD	GH9	63	13	4.9
15	Cthe_3079	Orf2p	n/a	1515	338	4.5
16	Cthe_2089	CelS	GH48	3848	864	4.5
17	Cthe_0043	CelN	GH9, CBM3	122	28	4.4
18	Cthe_2147	CelO	GH5, CBM3	63	15	4.3
19	Cthe_0452	OlpC	n/a	140	34	4.1
20	Cthe_0661	Ricin B lectin	GH43, CBM13	62	17	3.8
21	Cthe_2549	Dockerin type I cellulosome prot	n/a	27	7	3.7
22	Cthe_2811	ManA	GH26, CBM	517	153	3.4
23	Cthe_1806	Cellulosome prot, dockerin type I	Dockerin type I	95	29	3.3
24	Cthe_0405	CelL	GH5	96	29	3.3
25	Cthe_0246	PL11	PL11, CBM6	119	36	3.3
26	Cthe_2950	PL1	PL1, CBM35	49	15	3.2
27	Cthe_2271	Dockerin type I cellulosome prot	n/a	60	19	3.2
28	Cthe_0745	CelW	GH9, CBM3	124	40	3.1
29	Cthe_3141	Lipolytic enzyme, G-D-S-L	CE12, CBM6	23	8	2.9
30	Cthe_1400	GH53	GH53	31	11	2.8
31	Cthe_0736	Cellulosome anchoring prot, cohesin region	n/a	122	43	2.8
32	Cthe_0536	CelB	GH5	972	352	2.8
33	Cthe_0640	Cellulosome prot, dockerin type I	n/a	50	18	2.7
34	Cthe_2761	GH9	GH9, CBM3	44	16	2.7
35	Cthe_0032	GH26	GH26	423	159	2.7
36	Cthe_2195	CBM6	CBM6	4	2	2.5
37	Cthe_2138	GH43	GH43, CBM42	13	5	2.4
38	Cthe_0258	Cellulosome prot, dockerin type I	n/a	73	31	2.3
39	Cthe_2196	GH43	GH43, CBM6	2	1	2.3
40	Cthe_3012	GH30	GH30, CBM6	106	47	2.3
41	Cthe_2139	GH30, GH43, GH54	GH30, GH43, GH54	13	6	2.3
42	Cthe_2590	XynD	GH10, CBM22	148	67	2.2
43	Cthe_0015	Alpha-L-arabinofuranosidase B	GH43, GH54	203	92	2.2
44	Cthe_2038	Cellulosome prot, dockerin type I	n/a	48	22	2.1
45	Cthe_1307	SdbA	n/a	207	101	2.1
46	Cthe_2197	GH2	GH2, CBM6	2	1	2.0
47	Cthe_0435	Cel124A	GH124	401	205	2.0
**30 GENES THAT DEFINED AS UNCHANGED (2 > FC > 0.5)**						
48	Cthe_1838	XynC	GH10, CBM22	746	405	1.8
49	Cthe_0191	Proteinase inhibitor I4, serpin	n/a	16	9	1.8
50	Cthe_2194	CE1	CE1, CBM6	12	7	1.8
51	Cthe_2760	CelV	GH9, CBM3	162	92	1.8
52	Cthe_0190	Proteinase inhibitor I4, serpin	n/a	20	12	1.8
53	Cthe_3136	Peptidase S8/S53 subtilisin kexin sedolisin	n/a	36	22	1.7
54	Cthe_0269	CelA	GH8	1885	1136	1.7
55	Cthe_0274	CelP	GH9	116	73	1.6
56	Cthe_0543	CelF	GH9, CBM3	270	175	1.5
57	Cthe_1271	GH43	GH43, CBM6	168	116	1.5
58	Cthe_1963	XynZ	GH10, CE1, CBM6	532	390	1.4
59	Cthe_0109	Cellulosome prot, dockerin type I	n/a	24	18	1.3
60	Cthe_0821	Coagulation factor 5/8 type-like	GH5	1694	1266	1.3
61	Cthe_2949	Pectinesterase	CE8	10	8	1.3
62	Cthe_2812	CelT	GH9	342	291	1.2
63	Cthe_0729	Cellulosome prot, dockerin type I	n/a	66	59	1.1
64	Cthe_0735	Cellulosome anchoring prot, cohesin region	n/a	141	127	1.1
65	Cthe_0578	CelR	GH9, CBM3	238	216	1.1
66	Cthe_2179	Pectate lyase/Amb allergen	PL1, PL9, CBM35	23	22	1.1
67	Cthe_0433	GH9	GH9, CBM3	308	306	1.0
68	Cthe_0918	Cellulosome prot, dockerin type I	n/a	50	50	1.0
69	Cthe_0270	ChiA	GH18	85	92	0.9
70	Cthe_2972	XynA/U	GH11, CE4, CBM4	1212	1357	0.9
71	Cthe_2137	Cellulosome prot, dockerin type I	GH39, CBM35	7	8	0.9
72	Cthe_0624	CelJ	GH9, GH44, CBM30	93	116	0.8
73	Cthe_3132	Cellulosome prot, dockerin type I	n/a	63	79	0.8
74	Cthe_0625	CelQ	GH9, CBM3	190	248	0.8
75	Cthe_2879	Cellulosome prot, dockerin type I	CE6	13	18	0.7
76	Cthe_0044	Cellulosome prot, dockerin type I	n/a	17	24	0.7
77	Cthe_0797	CelE	GH5, CE2	180	311	0.6
**3 DOWN-REGULATED GENES (FC ≤ 0.5); ALL STATISTICALLY SIGNIFICANT (P < 0.05)**						
78	Cthe_2193	GH5	GH5, CBM6	67	135	0.5
79	Cthe_2360	CelU	GH9, CBM3	13	32	0.4
80	Cthe_1398	XghA	GH74	989	2817	0.4
**1 GENE DETECTED IN CELLOBIOSE-GROWN CELLS ONLY (CBO)**						
81	Cthe_0438	Cellulosome prot, dockerin type I	n/a	0	4	
**Average FC value for all genes (except the CBO)**						3.5

*The table is sorted in the order of FC values. The glycoside hydrolase (GH) and carbohydrate-binding module (CBM) families were determined according to the annotations in GenBank and the CAZY database (www.CAZY.org). Other abbreviations: Cbh, cellobiohydrolase; CBO, transcript that detected only in cellobiose-grown cells; CE, carbohydrate esterases; Cel, cellulase; Chi, chitinase; CipA, cellulose-integrating protein A; Lic, lichinase; Man, mannanase; n/a, not applicable; OlpB, outer layer protein; ORF2p, open reading frame 2p; PL, polysaccharide lyase; Prot, protein; SdbA, scaffoldin-dockerin binding component A; XghA, xyloglucanase Xgh74A; Xyn, xylanase*.

Analysis of this sub-transcriptome showed the following features: first, the overall cellulosome-associated genes were up-regulated significantly in PYP- vs. cellobiose-grown cells, reflected by the facts that out of the 81 cellulosomal genes, 47 (i.e., 58%) were up-regulated with *FC* ≥ 2.0. In contrast, only 4 out of 81 (i.e., about 5%) cellulosomal genes, including a GH5 gene (Cthe_2193), CelU (Cthe_2360), XghA (Cthe_1398), and Cthe_0438, were down-regulated by two-fold or detected in celllobiose-grown cells only. The overall average FC value for all these 81 cellulosomal genes is 3.5 (see the bottom row in Table 2), suggesting the whole cellulosome machinery was “geared up” at the late stationary phase on PYP substrate.

Secondly, the primary scaffoldin, CipA, and the main secondary scaffoldins, OlpB, Orf2P, and SdbA, have shown significant up-regulation and were found to have the highest abundance on the transcriptional level. This implies that the cellulosomal system is crucial for the efficient degradation of pretreated PYP. Our data further verifies the notion that the *C. thermocellum* cellulosome is the main contributor to the extremely high activity observed in cellulose degradation. Additionally, one putative scaffoldin gene, Cthe_0736 (cellulosome anchoring protein) has been up-regulated as much as 2.8 times, i.e., FC 2.8 (Table 2, row 31). OlpC, which has been recently identified as an important outer layer protein - cellulosome anchoring protein cohesin subunit (Pinheiro et al., 2009), was also found to be up-regulated in this study (Cthe_0452, FC 4.1; Table 2, row 19).

Thirdly, the major cellulosomal cellulases, such as CelS (Cthe_2089, exo-, FC 4.5; RPKM 3848—the most abundant cellulosomal transcripts in PYP-grown cells), CelA (Cthe_0269, endo-, FC 1.7, RPKM 1885—the third most abundant transcript in PYP-grown cells), CbhA (Cthe_0413, exo/endo, FC 7.5, RPKM 367), and Cel124A (Cthe_0435, endo-; FC 2.0; RPKM 401) were remarkably up-regulated, as shown in Table 2. Exo- and endo- refer to the substrate site upon which the GHs act. While exo-cellulases remove one or more sugar units from the ends of polysaccharide chain, endoglucanases randomly hydrolyze the internal glycosidic bonds of polysaccharides. Among the above listed enzymes, CelS was reported to display high synergy with the endo-Cel124A (Brás et al., 2011). Our observation that the CelS had the highest RPKM value in PYP-grown cells is consistent with a report that a knockout mutant of this gene showed a ~60% reduction in cell cellulolytic performance (Olson et al., 2010), and is also consistent with a most recent finding that CelS gene was found to be highly expressed in *C. thermocellum* cells grown on both pretreated switchgrass and cottonwood substrates (Wilson et al., 2013a). This study furthered such transcriptional analysis by showing that the transcript of CelS is not only highly abundant, but also significantly responsive to biomass substrate, as its RPKM level in cellobiose-grown cell was 4.5 times lower (Table 2).

In addition, there was one cellulosome dockerin type I gene (Cthe_0438) for which the transcript was detected only in cellobiose-grown cells, with a low RPKM value of 4 (Table 2, row 81). This observation is consistent with a previous report regarding its absence in the sub-proteome of cellulosomes from cells grown on pretreated switchgrass (Raman et al., 2009). The domain architecture of Cthe_0438 is “DUF843-type I dockerin” (DUF indicates domain of unknown function). It is difficult to classify the association of this gene with any of the specific catalytic enzyme types, cellulases or hemicellulases, and thus this issue may warrant further studies.

### Exploring the correlation between RNA-Seq and published proteomic data for cellulosomal genes

**Figure 5:**
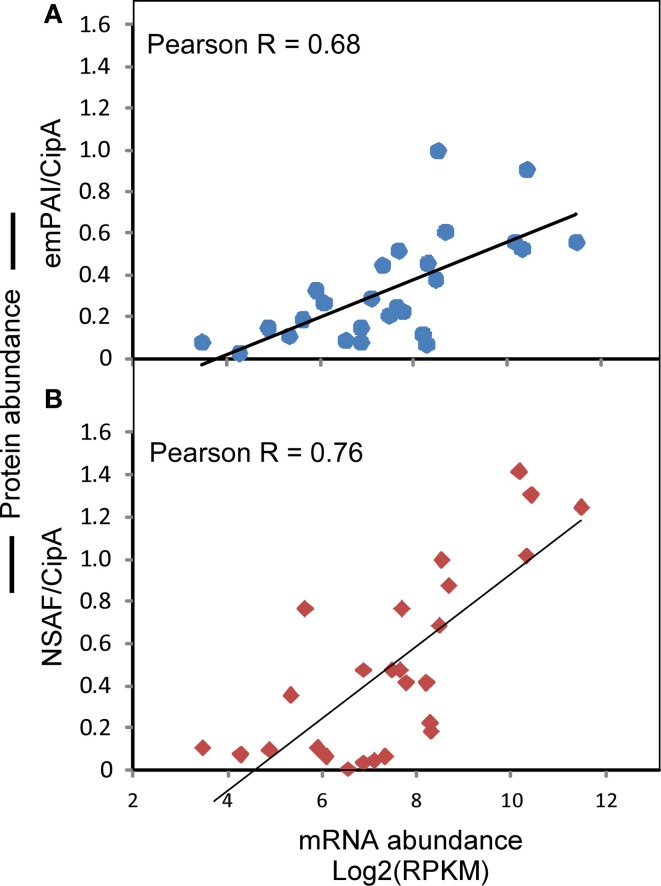
**Correlation between the observed gene abundances in this study and the published protein abundance data of cellulosomal components. (A)** Correlation between Log2(RPKM) and emPAI/CipA. **(B)** Correlation between Log2(RPKM) and NSAF/CipA. The literature sources for data of emPAI/CipA and NSAF/CipA are described in the Results and Discussion section.

Literature reports have described the quantitative sub-proteomic data of cellulosomes extracted from cell-free culture filtrates of *C. thermocellum* grown to late stationary phase on cellobiose, Avicel, and other cellulosic substrates (Gold and Martin, 2007; Raman et al., 2009). In one study (Gold and Martin, 2007), an “emPAI,” defined as the exponentially modified protein abundance index, showed a linear relationship with protein concentration and was normalized to the value obtained for CipA. Similarly, in another study (Raman et al., 2009), the normalized spectral abundance factor (NSAF) represented the number of spectral counts divided by the number of amino acid residues in the protein and was also normalized to the value obtained for CipA. To determine whether or not a correlative relationship exists between these published cellulosomal sub-proteomic data and the RNA-Seq RPKM data in the present study, we first retrieved the emPAI/CipA and *NSAF/CipA* data from the literature (Supplementary Data Sheet 3, section 2), and then plotted it against the Log2(RPKM) data for the genes encoding the same proteins in this study. The results showed that despite the fact that the plotted data were obtained from three different research groups, there remain strong correlations between the RNA-Seq RPKM and the protein abundance indicators of emPAI/CipA and NSAF/CipA, with Pearson correlation coefficient values being 0.68 and 0.76, respectively (Figures 5A,B).

### Non-cellulosomal glycoside hydrolase proteins and “free” cellulases

In addition to the GHs existing in cellulosomes, the *C. thermocellum* genome includes non-cellulosomal genes coding for GHs, among which 12 were up-regulated by more than two-fold (Table 3). Notably, both CelC (Cthe_2807) and Lic16A (Cthe_2809), the two members in the putative CelC- GlyR3-Lic16A operon (Newcomb et al., 2011), were up-regulated and predicted to be extracellular and/or bacterial cell wall associated (Table 3). This was consistent with a recent report that genes in this operon were mainly expressed in the stationary phase (very little during exponential phase) of pure cellulose (Avicel) fermentation (Raman et al., 2011). In addition, Lic16A contains CBM54 and a tandem of four CBM4s, in which both types of CBMs are able to bind xylan; as well as cellulose (Dvortsov et al., 2010). Remarkably, the grouping of four CBM4s in this protein has an ~100-fold higher binding constant for xylan and cellulose than that of a protein with a single CBM4 module (Dvortsov et al., 2012).

**Table 3:** **Twelve non-cellulosomal GH genes that were up-regulated by two or more folds in pretreated yellow poplar (PYP)- compared with cellobiose-grown *C. thermocellum* cells**.

**Gene ID**	**Protein and GH family^a^**	***FC***	**Cellular location prediction^b^**
Cthe_1256	GH3; bglB; β-glucosidase B	28.6	n/a
Cthe_1428	GH1; bgl	9.1	Cytoplasmic (Psortb)
Cthe_2744	GH23; lytic transglycosylase	7.5	Membrane (UniProt)
Cthe_2809	GH16, CBM4, CBM54; β-1,3-1,4-glucanase, Lic16A	6.0	Cell wall (Psortb) Extracellular (Psortb)
Cthe_1471	GH5; Rsi24C	5.9	Membrane (Bahari et al., 2011)
Cthe_2807	GH5; CelC, endoglucanase C	5.0	Extracellular (Psortb)
Cthe_0043	GH9, CBM3; CelN, Cel9N	4.4	Membrane (Psortb)
Cthe_0322	GH3	4.3	Extracellular (Uniprot)
Cthe_2191	α-glucan branching enzyme; GH13	3.3	Membrane (Burton, 2010)
Cthe_2548	GH51, α-N-arabinofuranosidase	3.3	Cytoplasmic (UniProt)
Cthe_1221	GH94, GT84; glycosyltransferase	3.0	Membrane (Psortb)
Cthe_0795	GH13, CBM34; α-amylase, catalytic region	2.2	Cytoplasmic (Psortb)

a *GH family characterization was based on CAZy database (http://www.cazy.org/)*.

b The cellular location was based on three sources: literature, UniProt database

*(http://www.uniprot.org/), or prediction by Psortb program (http://www.psort.org/psortb/index.html). All listed genes were statistically significantly up-regulated (p < 0.05). n/a, not available*.

It is surprising that Cel9I (Cthe_0040), an important non-cellulosomal processive endoglucanase that could digest crystalline cellulose with high efficiency, showed low abundance and FC value (RPKM 62 in PYP-grown cells, FC 1.3; see Supplementary Data Sheet 1, row no. 1875). Similarly, the only non-cellulosomal exo-cellulase CelY (Cthe_0071, exo-) also showed both low abundance and FC value (RPKM 61, FC 1.4, see Supplementary Data Sheet 1, row no. 1741), which is consistent with a recent report that knocking out the CelY gene had no significant impact on the cellulolytic capacity of the strain (Olson et al., 2010). Based on the evidence above, it would seem that the free-enzyme system plays a less important role than does the cellulosome system in the degradation of PYP by *C. thermocellum*.

### Responsive sigma factors coupled with membrane-associated anti-sigma factors as a mechanism for biosensing biomass substrates

Because COG analysis had revealed that the category of “signal transduction mechanisms, [T]” was among the most represented categories in the transcriptome of PYP-grown cells, we checked the genes related to the signal transmission attributed to sensing cellulose and other polysaccharide substrates. Recently, it was proposed the possible role of seven membrane-associated RsgI-like anti-sigma factors (referred to as RsgI) and their sigma factor partners (referred as SigI) in extracellular carbohydrate-sensing and glycosidase gene regulation in *C. thermocellum* (Kahel-Raifer et al., 2010; Nataf et al., 2010). In their proposed model, RsgI senses the presence of cellulose and other biomass components in the extracellular medium via its CBM domains, whereas SigI mediates the intracellular activation of different glycosidase genes (Kahel-Raifer et al., 2010; Nataf et al., 2010; Bahari et al., 2011). These predicted gene pairs include: SigI1- RsgI1 (Cthe_0058- Cthe_0059), σI2-RsgI2 (Cthe_0268- Cthe_0267), σI3-RsgI3 (Cthe_0315- Cthe_0316), σI4-RsgI4 (Cthe_0403- Cthe_0404), σI5-RsgI5 (Cthe_1272- Cthe_1273), σI6-RsgI6 (Cthe_2120- Cthe_2119), and σ24C-Rsi24C (Cthe_1470- Cthe_1471). Among them, three SigI genes (Cthe_0058, Cthe_0268, Cthe_0403) were up-regulated during later stages of pure cellulose (Avicel) fermentation (Raman et al., 2011).

In this study, while the transcripts of all seven pairs of genes had been detected (see Supplementary Data Sheet 1), three pairs of genes and an extra SigI-RsgI pair (which was not included in the initial literature prediction shown above) were up-regulated with FC values above 3.0 in the PYP- against cellobiose-grown cells at the late stationary phase, as described below:

1. σI3-RsgI3 (Cthe_0315- Cthe_0316), with FC 4.2, and 5.9, respectively. According to literature, RsgI3 contains a PA14 dyad domain that target pectin (Nataf et al., 2010). The differential expression of this pair was not previously reported in cellulose-grown *C. thermocellum*.
2. σI4-RsgI4 (Cthe_0403- Cthe_0404), with FC 3.1, and 3.9, respectively. RsgI4 contains CBM3 that binds cellulose. This observation supports previous observation of this pairs being involved in the time course of pure cellulose fermentation by *C. thermocellum* (Raman et al., 2011).
3. σ^24C^-Rsi24C (Cthe_1470- Cthe_1470), with FC 4.0 and 5.9, respectively. Note that Rsi24C contains a module that resembles GH5 that target cellulose based on literature (Nataf et al., 2010). Similar to σI3-RsgI3, the differential expression of this pair was also not previously reported in cellulose-grown *C. thermocellum*.
4. Equally interesting, we found that another SigI-RsgI pair (Cthe_2521-Cthe_2522) was detected on transcriptome, with FC values of 3.9 and 4.8, respectively (Supplementary Data Sheet 1). The protein of sigma factor Cthe_2521 had been detected in the proteome of *C. thermocellum* cultured on cellobiose (Rydzak et al., 2012), but was not in the initial 7 pairs of SigI-RsgI proposed for polysaccharide signal transmission in the literature (Nataf et al., 2010). The possible role of this pair warrants further investigation.

### Genes related to cellodextrin transport and phosphorylation

**Figure 6:**
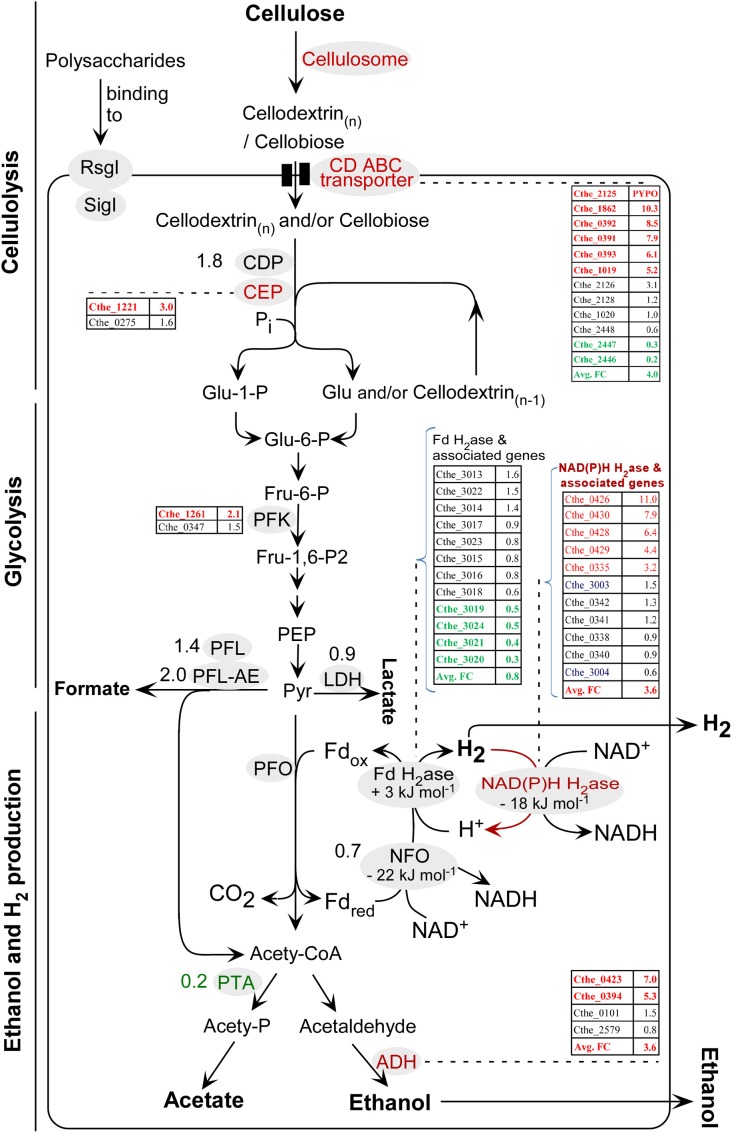
**Diagram for the primary steps in the conversion of cellulose to fermentation products using *C. thermocellum* ATCC 27405**. SigI-RsgI (sigma factors coupled with membrane-associated RsgI-like anti-sigma factors) are also illustrated in this diagram as they have been proposed in literature for polysaccharide triggered signal transmission in regulation of GH family genes. Detailed nomenclature of enzymes and their associated gene IDs can be found in Supplementary Data Sheet 3, section 3 and Table 4. For enzymes associated with a single gene ID, the gene ID is not included in the figure; instead, its fold change (FC) value is indicated directly beside the enzyme. For enzymes associated with multiple gene IDs, the gene IDs and their FC values are illustrated in sorted lists in the diagram, in order of either their FC values or locus IDs. Text in red and green represents the up-regulated and down-regulated genes, respectively, in PYP- against cellobiose-grown cells; in contrast, text in black represents the genes with no significant transcriptional changes between the two types of cells, i.e., 2.0 > *FC* value > 0.5. Nomenclature of metabolites: CoA, coenzyme A; ECH, energy conserving hydrogenase; Fd, ferredoxin; Fru, fructose; Glu, glucose; ox, oxidized; P, phosphate; PEP, phosphoenolpyruvate; Pi, inorganic phosphate; PYPO, transcript that was detected in PYP-grown cells only; Pyr, pyruvate; red, reduced.

*C. thermocellum* has been reported to use ABC-type transporters for uptake of oligosaccharides derived from cellulose hydrolysis (Strobel et al., 1995), which is an important energy-conserving mechanism by which importing long cellodextrins can reduce the cost of transport as one-ATP molecule is consumed per transport event. So far, four cellodextrin ABC transporters (carbohydrate-binding protein CbpA, B, C, and D) have been characterized for their substrate binding features (Nataf et al., 2009). Whereas CbpA (Cthe_0393) binds only to cellotriose (G3), CbpB (Cthe_1020) binds to G2-G5 cellodextrins, and CbpC (Cthe_2128) and D (Cthe_2446) bind to G3-G5 cellodextrins (Nataf et al., 2009; Rydzak et al., 2012). Several transcripts of these annotated genes have been detected in the transcriptome of *C. thermocellum* (Raman et al., 2011; Riederer et al., 2011). Previously, six cellodextrin ABC transporter genes (Cthe_1862, Cthe_0391-0393, and 1019-1020, including CbpA and CbpB) were found to be expressed at high levels throughout the course of Avicel alone fermentation (Raman et al., 2011). Most recently, Cthe_0391-0393 were found to be highly expressed on both pretreated switchgrass and cottonwood substrates (Wilson et al., 2013a). This study furthered such transcriptional analysis by comparing the gene expression between PYP- and cellobiose-grown cells at late stationary phase. A total of 12 transcripts of cellodextrin ABC transporters have been detected (Supplementary Data Sheet 3, section 3; also Figure 6), among which, seven were up-regulated in PYP- against cellobiose-grown cells, with FC values in the range of 3.1–10.3, suggesting that the PYP-grown cells had a mechanism for enhancing the uptake and utilization of polysaccharides derived from biomass substrates.

For the subsequent phosphorolytic cleavage of the imported oligosaccharides and cellobiose, this study identified the transcripts of one cellodextrin phosphorylase (CDP, Cthe_2989) and two cellobiose phosphorylases (CEP, Cthe_0275 and Cthe_1221). Among these, the CEP Cthe_1221 was up-regulated with a FC value of 3.0 (Supplementary Data Sheet 3, section 3; also Figure 6), suggesting this gene may warrant further studies.

### Genes related to glycolysis, pyruvate catabolism, and end-product synthesis

The deduced pathway for cellulolysis, glycolysis, ethanol, and H_2_ production in *C. thermocellum* ATCC 27405 is illustrated in Figure 6, which is in accordance to accumulated literature findings (Demain et al., 2005; Rydzak et al., 2009, 2011; Riederer et al., 2011; Carere, 2013). The set of gene IDs for the enzymes involved in above pathway were retrieved from the KEGG PATHWAY database (http://www.genome.jp/kegg) (Kanehisa et al., 2008), and were shown in Supplementary Data Sheet 3, section 3 with their FC values in PYP- vs. cellobiose-grown cells. Out of the listed genes, 18 and 12 were significantly up- and down-regulated, as shown in red and green text in Supplementary Data Sheet 3, section 3, respectively.

For pyruvate catabolism and end-product synthesis, *C. thermocellum* may convert pyruvate into (1) formate via pyruvate formate lyase (PFL) and pyruvate formate lyase activating enzyme (PFL-AE), (2) lactate via lactate dehydrogenase (LDH), (3) CO_2_, Fd_red_, and acetyl-CoA, in which acetyl-CoA eventually leads to the production of acetate and ethanol, the Fd_red_ leads to the formation of hydrogen (Figure 6). The up-regulation of two out of four alcohol dehydrogenase (ADH) genes, namely Cthe_0423 (FC value 7.0) and Cthe_0394 (FC value 5.3), raise the average FC value for ADHs to 3.6. In contrast, the phosphotransacetylase (PTA; Cthe_1029), whose activity had been verified in *C. thermocellum* (Lamed and Zeikus, 1980), was significantly down-regulated in PYP- against cellobiose-grown cells, with an FC value of 0.2. These data suggest that based on mRNA profiling, carbon flux of acetyl-CoA may preferentially be channeled to ethanol production in PYP-grown cells. The observed decrease of acetate/ethanol ratio, from 1.08 in cellobiose-grown cells to 0.92 in PYP-grown cells (Table 1), supports this carbon shift at the late stationary phase of PYP-grown cells.

Lactate and formate were not monitored in this study as previous study has shown that in the late growth phase of *C. thermocellum* cultures grown on cellobiose substrate, lactate, and formate together represent only a small fraction of the total end products produced (Islam et al., 2006). Future studies should also monitor the production of lactate, formate and CO_2_, which could provide a whole picture for carbon balance for the cells utilizing biomass substrates.

### Dynamics for H_2_ production with PYP as substrate

*C. thermocellum* genes encoding putative hydrogenases and sensory hydrogenases using ferredoxin and NAD(P)H as electron carriers are listed Table 4. In addition, hydrogenase maturation proteins are also listed. The classification of the above hydrogenases are mainly based on literatures that systematically characterized the putative hydrogenases in *C. thermocellum* and other species (Schut and Adams, 2009; Carere et al., 2012). Briefly, there are two types of hydrogenases (H_2_ases) according to the metal content in the respective active sites:

**Table 4:**
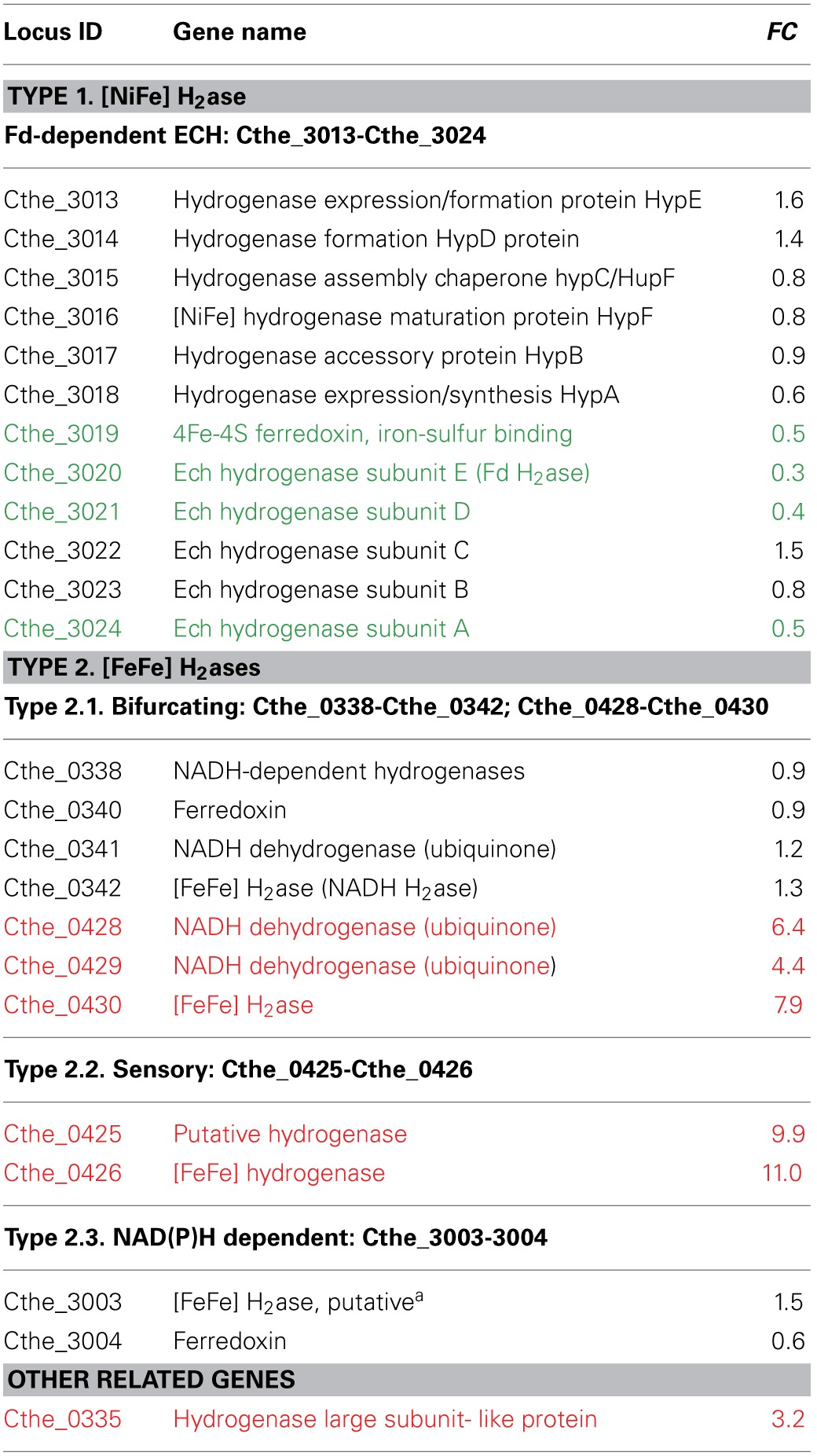
***C. thermocellum* genes encoding putative hydrogenases, sensory hydrogenases, and NADH:Fd oxidoreductases using ferredoxin and NAD(P)H as electron carriers**.

*In addition, hydrogenase maturation proteins are also listed. The table is arranged according to the hydrogenase classification described by two literature sources, as cited in the related Results and Discussion section; within each sub-category, it was arranged in order of locus IDs. For each gene, fold change (FC) value was calculated by dividing the RPKM of PYP cells by RPKM of cellobiose cells, as listed in Supplementary Data Sheet 1. RPKM, reads per kilobase of exon model per million mapped reads. Text in black represents the genes with no significant transcriptional changes between the two types of cells, i.e., 2.0 > FC value > 0.5. Text in red and green represents the genes that found to be significantly up-regulated and down-regulated, respectively, in PYP- against cellobiose-grown cells, based on statistic analysis in Supplementary Data Sheet 2. ECH, energy conserving hydrogenase. PYPO, transcript that detected in PYP-grown cells only*.

*^a^Cthe_3003 ([FeFe] H_2_ase, putative) was characterized as Fd-linked by Schut and Adams (2009), but as NAD(P)H dependent recently by Carere et al. (2012)*.

The first type is [NiFe] H_2_ase, a putative Fd-dependent energy converting hydrogenase (ECH). Its hexameric structural subunits are encoded by Cthe_3019-Cthe_3024, with assembly of its active site assisted by a suite of maturation proteins encoded by Cthe_3013-Cthe_3018 (Table 4, Figure 6). This study showed that the FC values for 7 of the 12 detected Fd-H_2_ase and related genes (Cthe_3013-Cthe_3015, Cthe_3017-Cthe_3018, and Cthe_3022-Cthe_3023) were in the range of 0.6–1.6, suggesting an unchanged status at the transcriptional level for these genes. However, the remaining five genes (Cthe_3019-Cthe_3021, Cthe_3023-Cthe_3024) encoding the Fd-H_2_ase structural subunits were significantly down-regulated with FC values between 0.3 and 0.5, which might account for the observed lower H_2_ yield in PYP-grown cells, compared to the control cellobiose-grown cells. This finding implies that the [NiFe] ECH H_2_ase likely functions in the H_2_ production direction since its down-regulation led to less H_2_ accumulation in the culture headspace.

The second type is [FeFe] H_2_ases that include two bifurcating H_2_ases: both being trimeric, and encoded by Cthe_0338-Cthe_0342 and Cthe_0428-Cthe_0430, respectively. Only seven genes are listed in Table 4 because Cthe_0339 is annotated as histidine kinase in GenBank protein database. The second type also includes a sensory H_2_ase (Cthe_0425-Cthe_0426) and a NAD(P)H dependent H_2_ase (Cthe_3003-Cthe_3004), as listed in Table 4. The data showed that the FC values for the trimeric bifurcating H_2_ase genes (Cthe_0338, Cthe_0340-Cthe_0342) and the NAD(P)H dependent H_2_ase genes (Cthe_3003-3004) were in the range of 0.6–1.5, indicating an unchanged status for their transcription (Figure 6). A homolog of this trimeric H_2_ase has been uncovered in *Acetobacterium woodii* which functions in H_2_ consumption yielding reduced Fd and NAD(P)H (Schut and Adams, 2009; Hess et al., 2013a,b). In contrast, genes encoding the trimeric bifurcating H_2_ase (Cthe_0428-Cthe_0430) were significantly up-regulated in PYP-grown cells, with FC values of 6.4–7.9 (Figure 6). Based on literature (Schut and Adams, 2009; Calusinska et al., 2010), the trimeric [FeFe] H_2_ase identified in *C. thermocellum* is a putative bifurcating hydrogenase. This cytoplasmic enzyme was initially characterized in *Thermotoga maritima* and uses both reduced ferredoxin and NAD(P)H as substrates, and functions in H_2_ production (Schut and Adams, 2009; Hess et al., 2013a). Yet the up-regulation of Cthe_0428-Cthe_0430 (this work) led to decreased H_2_ production when cultured in PYP- vs. cellobiose-grown cells. As such, the exact role of the *C. thermocellum* trimeric H_2_ase is unknown, which makes it hard to link the above transcriptional data to the observed lower H_2_ yield in PYP-grown cells. Further studies are warranted to investigate the exact direction (production or consumption of H_2_) for each of the above [FeFe] H_2_ases.

In addition, the sensory H_2_ase encoded by Cthe_0425-Cthe_0426 had a FC value of 9.9 and 11.0, respectively (Table 4), and future studies are also needed to explore the implication of the significant up-regulation of these two genes in PYP-grown cells.

### Genes for degrading hemicellulose and lignin in feedstock biomass

We noticed that some important hemicellulase genes, such as XynY (Cthe_0912, FC 13.8), XynD (Cthe_2590, FC 2.2), XynZ (Cthe_1963, FC 1.4), ManA (Cthe_2811, FC 3.4) were up-regulated in PYP- against cellobiose-grown cells (Table 2; cellulosomal proteins). In addition, some auxiliary enzymes such as α-N-arabinofuranosidase (Cthe_2548, FC 3.3; Table 3, non-cellulosomal protein) were also up-regulated. This is probably caused by the adaptation of the strain to digest the PYP, of which the chemical composition and structure are much different from those of cellobiose. In contrast, we did not identify the primary genes involved in lignin modification and degradation, such as laccases, lignin peroxidases, or manganese peroxidases.

### Growth phase and the transcriptome of cells

The main focus of this study was to investigate the effects of different carbon sources (PYP vs. cellobiose) on the transcriptome of the cells. To attain this goal, the selection of sampling point was crucial. The experimental design of this study in choosing the late stationary phase is in accordance with literature (Gold and Martin, 2007; Raman et al., 2009). One merit for choosing this sampling point is that our transcriptomic data can be cross-analyzed with the literature's protein data of cellulosomes extracted from cellobiose-grown cells, which are used as one means of validating the transcriptomic data in this study.

Another merit in choosing this sampling point is that compared with other early growth stages (exponential or early stationary phases), the levels of simple, soluble sugars in PYP- and cellobiose-grown culture are both very low: due to either the limit of bacterial enzymes in releasing sugars from PYP or the depletion of cellobiose, respectively. In this sense, late stationary phase is relatively more suitable phase than other phases for comparing transcriptomes derived from different carbon substrate-grown cells in this study.

Nevertheless, it is noteworthy that different growth phases may cause some shifts to some metabolic pathways; thus future studies comparing cells grown on cellobiose vs. cells grown on woody biomass at the exponential phase would provide another angle to investigate the effects of biomass substrate on the transcriptomes of *C. thermocellum* cells, which may lead to the identification of both growth phase- and biomass substrate-responsive genes. In addition, the transcriptional profiles of these two cell samples could also be affected by the distinct concentrations of carbohydrate sources as well as the residual concentrations of metabolites/nutrients present at time of sampling, which will remain a challenge for future studies in managing these variables—some of which are nearly uncontrollable.

## Conclusions

We conducted a RNA-Seq analysis of the transcriptional profiles of *C. thermocellum* grown on PYP and cellobiose, which is different from previous transcriptional studies that focused on degrading cellulose alone substrates, or sub-proteomic studies that focused on cellulosomal protein components. We found that nearly 60% of the genes encoding the protein components of the cellulosomes—the core machinery for cellulose degradation—were up-regulated, whereas only 5% were down-regulated. The top up-regulated cellulosomal genes, along with the responsive SigI-RsgI and cellodextrin transporter genes, present promising candidate genes for engineering *C. thermocellum* strains to improve their capacity in effectively converting lignocellulosic biomass substrates. Furthermore, the identified differentially expressed NAD(P)H H_2_ase, ADH, and PTA genes may provide insight into how the cells regulate the production of H_2_ and ethanol under the carbon-limited condition.

### Conflict of interest statement

The authors declare that the research was conducted in the absence of any commercial or financial relationships that could be construed as a potential conflict of interest.

## Supplementary material

Supplementary Data Sheet 1 **List of 3081 genes with their RPKM and fold change values determined in PYP- vs. cellobiose-grown *C. thermocellum* cells**.

Supplementary Data Sheet 2 **List of 1211 up-regulated and 314 down-regulated genes in PYP-grown *C. thermocellum* cells**.

Supplementary Data Sheet 3 **Section 1**. Forward (F) and reverse (R) primer sequences for quantitative reverse transcription-PCR (qRT-PCR) analysis of selected genes in *C. thermocellum*. **Section 2**. RPKM values of cellulosomal genes based on this study and their protein abundance data retrieved from literature for cellobiose-grown *C. thermocellum* cells at the late stationary phase. **Section 3**. Fold change values for genes related to cellodextrin transport and phosphorylation, glycolysis, pyruvate catabolism and end-product synthesis in *C. thermocellum*.

## Abbreviations

Abf: α-N-arabinofuranosidase
ADH: alcohol dehydrogenase
CBO: cellobiose-grown cells only
Cbp: carbohydrate-binding protein
CBP: consolidated bioprocessing
CDP: cellodextrin phosphorylase
CEP: cellobiose phosphorylases
COG: clusters of orthologous groups
*emPAI*: *exponentially modified protein abundance index*
*FC*: fold changes
Fdred: reduced ferredoxin
GH: glycoside hydrolases
NSAF: normalized spectral abundance factor
*PF*: *passing filter*
PTA: phosphotransacetylase
PYP: pretreated yellow poplar
PYPO: PYP-grown cells only
*qRT-PCR*: *quantitative reverse transcription-PCR*
RPKM: reads per kilobase of exon model per million mapped reads
RsgI: RsgI-like anti-sigma factors
SigI: sigma factor.

## References

[B1] Alper H. Stephanopoulos G. (2009). Engineering for biofuels: exploiting innate microbial capacity or importing biosynthetic potential? Nat. Rev. Microbiol. 7, 715–723 10.1038/nrmicro2186 19756010

[B2] Bahari L. Gilad Y. Borovok I. Kahel-Raifer H. Dassa B. Nataf Y. (2011). Glycoside hydrolases as components of putative carbohydrate biosensor proteins in *Clostridium thermocellum*. J. Ind. Microbiol. Biotechnol. 38, 825–832 10.1007/s10295-010-0848-9 20820855

[B3] Baker J. O. Vinzant T. B. Ehrman C. I. Adney W. S. Himmel M. E. (1997). Use of a new membrane-reactor saccharification assay to evaluate the performance of celluloses under simulated ssf conditions. Appl. Biochem. Biotechnol. 63, 585–595 10.1007/BF02920456 18576113

[B4] Bayer E. A. Belaich J. P. Shoham Y. Lamed R. (2004). The cellulosomes: multienzyme machines for degradation of plant cell wall polysaccharides. Annu. Rev. Microbiol. 58, 521–554 10.1146/annurev.micro.57.030502.091022 15487947

[B5] Bayer E. A. Chanzy H. Lamed R. Shoham Y. (1998). Cellulose, cellulases and cellulosomes. Curr. Opin. Struct. Biol. 8, 548–557 10.1016/S0959-440X(98)80143-7 9818257

[B6] Bayer E. A. Lamed R. Himmel M. E. (2007). The potential of cellulases and cellulosomes for cellulosic waste management. Curr. Opin. Biotechnol. 18, 237–245 10.1016/j.copbio.2007.04.004 17462879

[B7] Brás J. L. Cartmell A. Carvalho A. L. M. Verzé G. Bayer E. A. Vazana Y. (2011). Structural insights into a unique cellulase fold and mechanism of cellulose hydrolysis. Proc. Natl. Acad. Sci. U.S.A. 108, 5237–5242 10.1073/pnas.1015006108 21393568 PMC3069175

[B8] Brown S. D. Lamed R. Morag E. Borovok I. Shoham Y. Klingeman D. M. (2012). Draft genome sequences for *Clostridium thermocellum* wild-type strain YS and derived cellulose adhesion-defective mutant strain AD2. J. Bacteriol. 194, 3290–3291 10.1128/JB.00473-12 22628515 PMC3370843

[B8a] Burton E. (2010). Proteomic Analysis of Cellulose Metabolism in Clostridium thermocellum. Master thesis, Concordia University, Montreal

[B9] Calusinska M. Happe T. Joris B. Wilmotte A. (2010). The surprising diversity of clostridial hydrogenases: a comparative genomic perspective. Microbiology 156, 1575–1588 10.1099/mic.0.032771-0 20395274

[B10] Carere C. R. Rydzak T. Verbeke T. J. Cicek N. Levin D. B. Sparling R. (2012). Linking genome content to biofuel production yields: a meta-analysis of major catabolic pathways among select H2 and ethanol-producing bacteria. BMC Microbiol. 12:295 10.1186/1471-2180-12-295 23249097 PMC3561251

[B11] Carere R. C. (2013). Genomics of Cellulolytic Clostridia and Development of Rational Metabolic Engineering Strategies. Ph.D., University of Manitoba

[B12] Chen Z. Duan X. (2011). Ribosomal RNA depletion for massively parallel bacterial RNA-sequencing applications. Methods Mol. Biol. 733, 93–103 10.1007/978-1-61779-089-8_7 21431765

[B13] Cooksley C. M. Zhang Y. Wang H. Redl S. Winzer K. Minton N. P. (2012). Targeted mutagenesis of the *Clostridium acetobutylicum* Acetone-Butanol-Ethanol fermentation pathway. Metab. Eng. 14, 630–641 10.1016/j.ymben.2012.09.001 22982601

[B14] Cusick K. Lee Y.-Y. Youchak B. Belas R. (2012). Perturbation of FliL interferes with Proteus mirabilis swarmer cell gene expression and differentiation. J. Bacteriol. 194, 437–447 10.1128/JB.05998-11 22081397 PMC3256649

[B15] Demain A. L. Newcomb M. Wu J. H. D. (2005). Cellulase, clostridia, and ethanol. Microbiol. Mol. Biol. Rev. 69, 124–154 10.1128/MMBR.69.1.124-154.2005 15755956 PMC1082790

[B16] Dror T. W. Rolider A. Bayer E. A. Lamed R. Shoham Y. (2005). Regulation of major cellulosomal endoglucanases of *Clostridium thermocellum* differs from that of a prominent cellulosomal xylanase. J. Bacteriol. 187, 2261–2266 10.1128/JB.187.7.2261-2266.2005 15774868 PMC1065243

[B17] Dvortsov I. Lunina N. Zverlov V. Velikodvorskaya G. (2010). Substrate-binding properties of the family 54 module of *Clostridium thermocellum* Lic16A laminarinase. Mol. Biol. 44, 591–595 10.1134/S002689331004014X 20873227

[B18] Dvortsov I. Lunina N. Zverlov V. Velikodvorskaya G. (2012). Properties of four C-terminal carbohydrate-binding modules (CBM4) of laminarinase Lic16A of *Clostridium thermocellum*. Mol. Biol. 46, 817–822 10.1134/S0026893312060039 23350238

[B19] Ferreira S. J. Senning M. Sonnewald S. Keßling P.-M. Goldstein R. Sonnewald U. (2010). Comparative transcriptome analysis coupled to X-ray CT reveals sucrose supply and growth velocity as major determinants of potato tuber starch biosynthesis. BMC Genomics 11:93 10.1186/1471-2164-11-93 20137087 PMC2827413

[B20] Gold N. Martin V. (2007). Global view of the *clostridium thermocellum* cellulosome revealed by quantitative proteomic analysis? J. Bacteriol. 189, 6787–6795 10.1128/JB.00882-07 17644599 PMC2045192

[B21] Hess V. González J. M. Parthasarathy A. Buckel W. Müller V. (2013a). Caffeate respiration in the acetogenic bacterium *Acetobacterium woodii*: a coenzyme A loop saves energy for caffeate activation. Appl. Environ. Microbiol. 79, 1942–1947 10.1128/AEM.03604-12 23315745 PMC3592220

[B22] Hess V. Schuchmann K. Müller V. (2013b). The ferredoxin: NAD+ oxidoreductase (Rnf) from the acetogen *Acetobacterium woodii* requires Na+ and is reversibly coupled to the membrane potential. J. Biol. Chem. 288, 31496–31502 10.1074/jbc.M113.510255 24045950 PMC3814746

[B23] Himmel M. E. Ding S. Y. Johnson D. K. Adney W. S. Nimlos M. R. Brady J. W. (2007). Biomass recalcitrance: engineering plants and enzymes for biofuels production. Science 315, 804–807 10.1126/science.1137016 17289988

[B24] Huang W. Nadeem A. Zhang B. Babar M. Soller M. Khatib H. (2012). Characterization and comparison of the leukocyte transcriptomes of three cattle breeds. PLoS ONE 7:e30244 10.1371/journal.pone.0030244 22291923 PMC3264571

[B24a] Irwin D. Leathers T. Greene R. Wilson D. (2003). Corn fiber hydrolysis by Thermobifida fusca extracellular enzymes. Appl. Environ. Microbiol. 61, 352–358 10.1007/s00253-002-1210-6 12743765

[B25] Islam R. Cicek N. Sparling R. Levin D. (2006). Effect of substrate loading on hydrogen production during anaerobic fermentation by *Clostridium thermocellum* 27405. Appl. Microbiol. Biotechnol. 72, 576–583 10.1007/s00253-006-0316-7 16685495

[B26] Ji H. Gheysen G. Denil S. Lindsey K. Topping J. F. Nahar K. (2013). Transcriptional analysis through RNA sequencing of giant cells induced by Meloidogyne graminicola in rice roots. J. Exp. Bot. 64, 3885–3898 10.1093/jxb/ert219 23881398 PMC3745741

[B27] Jia Y. Lisch D. R. Ohtsu K. Scanlon M. J. Nettleton D. Schnable P. S. (2009). Loss of RNA–dependent RNA polymerase 2 (RDR2) function causes widespread and unexpected changes in the expression of transposons, genes, and 24-nt small RNAs. PLoS Genet. 5:e1000737 10.1371/journal.pgen.1000737 19936292 PMC2774947

[B28] Kahel-Raifer H. Jindou S. Bahari L. Nataf Y. Shoham Y. Bayer E. A. (2010). The unique set of putative membrane-associated anti-σ factors in *Clostridium thermocellum* suggests a novel extracellular carbohydrate-sensing mechanism involved in gene regulation. FEMS Microbiol. Lett. 308, 84–93 10.1111/j.1574-6968.2010.01997.x 20487018

[B29] Kanehisa M. Araki M. Goto S. Hattori M. Hirakawa M. Itoh M. (2008). KEGG for linking genomes to life and the environment. Nucleic Acids Res. 36, D480–D484 10.1093/nar/gkm882 18077471 PMC2238879

[B30] Lamed R. Zeikus J. (1980). Ethanol production by thermophilic bacteria: relationship between fermentation product yields of and catabolic enzyme activities in *Clostridium thermocellum* and *Thermoanaerobium brockii*. J. Bacteriol. 144, 569–578 7430065 10.1128/jb.144.2.569-578.1980 PMC294704

[B31] Langmead B. Trapnell C. Pop M. Salzberg S. L. (2009). Ultrafast and memory-efficient alignment of short DNA sequences to the human genome. Genome Biol. 10:R25 10.1186/gb-2009-10-3-r25 19261174 PMC2690996

[B32] Lin C. Garrett A. S. De Kumar B. Smith E. R. Gogol M. Seidel C. (2011). Dynamic transcriptional events in embryonic stem cells mediated by the super elongation complex (SEC). Genes Dev. 25, 1486–1498 10.1101/gad.2059211 21764852 PMC3143939

[B33] Mortazavi A. Williams B. A. McCue K. Schaeffer L. Wold B. (2008). Mapping and quantifying mammalian transcriptomes by RNA-Seq. Nat. Methods 5, 621–628 10.1038/nmeth.1226 18516045 PMC13303166

[B34] Nagalakshmi U. Wang Z. Waern K. Shou C. Raha D. Gerstein M. (2008). The transcriptional landscape of the yeast genome defined by RNA sequencing. Science 320, 1344 10.1126/science.1158441 18451266 PMC2951732

[B35] Nataf Y. Bahari L. Kahel-Raifer H. Borovok I. Lamed R. Bayer E. A. (2010). *Clostridium thermocellum* cellulosomal genes are regulated by extracytoplasmic polysaccharides via alternative sigma factors. Proc. Natl. Acad Sc. U.S.A. 107, 18646–18651 10.1073/pnas.1012175107 20937888 PMC2972930

[B36] Nataf Y. Yaron S. Stahl F. Lamed R. Bayer E. A. Scheper T.-H. (2009). Cellodextrin and laminaribiose ABC transporters in *Clostridium thermocellum*. J. Bacteriol. 191, 203–209 10.1128/JB.01190-08 18952792 PMC2612431

[B37] Newcomb M. Millen J. Chen C.-Y. Wu J. D. (2011). Co-transcription of the celC gene cluster in *Clostridium thermocellum*. Appl. Microbiol. Biotechnol. 90, 625–634 10.1007/s00253-011-3121-x 21318364

[B38] Olson D. G. Tripathi S. A. Giannone R. J. Lo J. Caiazza N. C. Hogsett D. A. (2010). Deletion of the Cel48S cellulase from *Clostridium thermocellum*. Proc. Natl. Acad. Sci. U.S.A. 107, 17727–17732 10.1073/pnas.1003584107 20837514 PMC2955147

[B39] Pinheiro B. Gilbert H. Sakka K. Fernandes V. Prates J. Alves V. (2009). Functional insights into the role of novel type I cohesin and dockerin domains from *Clostridium thermocellum*. Biochem. J. 424, 375–384 10.1042/BJ20091152 19758121

[B40] Raman B. McKeown C. K. Rodriguez M. Brown S. D. Mielenz J. R. (2011). Transcriptomic analysis of *Clostridium thermocellum* ATCC 27405 cellulose fermentation. BMC Microbiol. 11:134 10.1186/1471-2180-11-134 21672225 PMC3130646

[B41] Raman B. Pan C. Hurst G. B. Rodriguez M. McKeown C. K. Lankford P. K. (2009). Impact of pretreated switchgrass and biomass carbohydrates on *clostridium thermocellum* ATCC 27405 cellulosome composition: a quantitative proteomic analysis. PLoS ONE 4:e5271 10.1371/journal.pone.0005271 19384422 PMC2668762

[B42] Riederer A. Takasuka T. E. Makino S.-I. Stevenson D. M. Bukhman Y. V. Elsen N. L. (2011). Global gene expression patterns in *Clostridium thermocellum* as determined by microarray analysis of chemostat cultures on cellulose or cellobiose. Appl. Environ. Microbiol. 77, 1243–1253 10.1128/AEM.02008-10 21169455 PMC3067202

[B43] Rydzak T. Levin D. B. Cicek N. Sparling R. (2009). Growth phase-dependant enzyme profile of pyruvate catabolism and end-product formation in *Clostridium thermocellum* ATCC 27405. J. Biotechnol. 140, 169–175 10.1016/j.jbiotec.2009.01.022 19428711

[B44] Rydzak T. Levin D. B. Cicek N. Sparling R. (2011). End-product induced metabolic shifts in *Clostridium thermocellum* ATCC 27405. Appl. Microbiol. Biotechnol. 92, 199–209 10.1007/s00253-011-3511-0 21837436

[B45] Rydzak T. McQueen P. Krokhin O. Spicer V. Ezzati P. Dwivedi R. (2012). Proteomic analysis of *Clostridium thermocellum* core metabolism: relative protein expression profiles and growth phase-dependent changes in protein expression. BMC Microbiol. 12:214 10.1186/1471-2180-12-214 22994686 PMC3492117

[B46] Schut G. J. Adams M. W. (2009). The iron-hydrogenase of Thermotoga maritima utilizes ferredoxin and NADH synergistically: a new perspective on anaerobic hydrogen production. J. Bacteriol. 191, 4451–4457 10.1128/JB.01582-08 19411328 PMC2698477

[B47] Stevenson D. Weimer P. (2005). Expression of 17 genes in *Clostridium thermocellum* ATCC 27405 during fermentation of cellulose or cellobiose in continuous culture. Appl. Environ. Microbiol. 71:4672 10.1128/AEM.71.8.4672-4678.2005 16085862 PMC1183361

[B48] Strobel H. Caldwell F. Dawson K. (1995). Carbohydrate transport by the anaerobic thermophile *Clostridium thermocellum* LQRI. Appl. Environ. Microbiol. 61, 4012–4015 16535164 10.1128/aem.61.11.4012-4015.1995 PMC1388600

[B49] Tang F. C. Barbacioru C. Wang Y. Z. Nordman E. Lee C. Xu N. L. (2009). mRNA-Seq whole-transcriptome analysis of a single cell. Nat. Methods 6, U377–U386 10.1038/nmeth.1315 19349980

[B50] Tatusov R. L. Galperin M. Y. Natale D. A. Koonin E. V. (2000). The COG database: a tool for genome-scale analysis of protein functions and evolution. Nucleic Acids Res. 28, 33–36 10.1093/nar/28.1.33 10592175 PMC102395

[B51] Templeton D. W. Scarlata C. J. Sluiter J. B. Wolfrum E. J. (2010). Compositional analysis of lignocellulosic feedstocks. 2. Method uncertainties. J. Agric. Food Chem. 58, 9054–9062 10.1021/jf100807b 20669952 PMC2923869

[B52] Tucker M. P. Farmer J. D. Keller F. A. Schell D. J. Nguyen Q. A. (1998). Comparison of yellow poplar pretreatment between NREL digester and sunds hydrolyzer. Appl. Biochem. Biotechnol. 70–72, 25–35 10.1007/BF02920121

[B53] van der Lelie D. Taghavi S. McCorkle S. M. Li L.-L. Malfatti S. A. Monteleone D. (2012). The metagenome of an anaerobic microbial community decomposing poplar wood chips. PLoS ONE 7:e36740 10.1371/journal.pone.0036740 22629327 PMC3357426

[B54] Vinzant T. B. Ponfick L. Nagle N. J. Ehrman C. I. Reynolds J. B. Himmel M. E. (1994). SSF comparison of selected woods from southern sawmills. Appl. Biochem. Biotechnol. 45, 611–626 10.1007/BF02941834

[B55] Wang Z. Gerstein M. Snyder M. (2009). RNA-Seq: a revolutionary tool for transcriptomics. Nature Reviews Genetics 10, 57–63 10.1038/nrg2484 19015660 PMC2949280

[B56] Wei H. Tucker M. P. Baker J. O. Harris M. Luo Y. Xu Q. (2012). Tracking dynamics of plant biomass composting by changes in substrate structure, microbial community, and enzyme activity. Biotechnol. Biofuels 5:20 10.1186/1754-6834-5-20 22490508 PMC3384452

[B57] Wei H. Xu Q. Taylor L. Baker J. Tucker M. Ding S. (2009). Natural paradigms of plant cell wall degradation. Curr. Opin. Biotechnol. 20, 330–338 10.1016/j.copbio.2009.05.008 19523812

[B58] Wilhelm B. T. Marguerat S. Watt S. Schubert F. Wood V. Goodhead I. (2008). Dynamic repertoire of a eukaryotic transcriptome surveyed at single-nucleotide resolution. Nature 453, 1239–1243 10.1038/nature07002 18488015

[B59] Wilson C. M. Rodriguez M. Jr. Johnson C. M. Martin S. L. Chu T. M. Wolfinger R. D. (2013a). Global transcriptome analysis of *Clostridium thermocellum* ATCC 27405 during growth on dilute acid pretreated Populus and switchgrass. Biotechnol. Biofuels 6:179 10.1186/1754-6834-6-179 24295562 PMC3880215

[B60] Wilson C. M. Yang S. Rodriguez M. Jr. Ma Q. Johnson C. M. Dice L. (2013b). *Clostridium thermocellum* transcriptomic profiles after exposure to furfural or heat stress. Biotechnol. Biofuels 6:131 10.1186/1754-6834-6-131 24028713 PMC3848806

[B61] Yang S. Giannone R. Dice L. Yang Z. Engle N. Tschaplinski T. (2012). *Clostridium thermocellum* ATCC27405 transcriptomic, metabolomic and proteomic profiles after ethanol stress. BMC Genomics 13:336 10.1186/1471-2164-13-336 22823947 PMC3478167

[B62] Yang S. Guarnieri M. T. Smolinski S. Ghirardi M. Pienkos P. T. (2013). De novo transcriptomic analysis of hydrogen production in the green alga Chlamydomonas moewusii through RNA-Seq. Biotechnol. Biofuels 6:118 10.1186/1754-6834-6-118 23971877 PMC3846465

[B63] Zverlov V. V. Kellermann J. Schwarz W. H. (2005). Functional subgenomics of *Clostridium thermocellum* cellulosomal genes: identification of the major catalytic components in the extracellular complex and detection of three new enzymes. Proteomics 5, 3646–3653 10.1002/pmic.200401199 16127726

[B64] Zverlov V. V. Schwarz W. H. (2006). The *Clostridium thermocellum* cellulosome: novel components and insights from the genomic sequence, in Cellulosome, eds Kataeva I. Ljungdahl L. (New York, NY: Nova Science Publishers), 119–151

