# Supplementary material for: Comparison of transcriptional profiles of Clostridium thermocellum grown on cellobiose and pretreated yellow poplar using RNA-Seq
Source: Front Microbiol. 2014 Apr 11;5:142. doi: 10.3389/fmicb.2014.00142 (PMC3990059; doi:10.3389/fmicb.2014.00142)
Supplement: Supplementary Data Sheet 3 — Section 1. Forward (F) and reverse (R) primer sequences for quantitative reverse transcription-PCR (qRT-PCR) analysis of selected genes in C. thermocellum. Section 2. RPKM values of cellulosomal genes based on this study and their protein abundance data retrieved from literature for cellobiose-grown C. thermocellum cells at the late stationary phase. Section 3. Fold change values for genes related to cellodextrin transport and phosphorylation, glycolysis, pyruvate catabolism and end-product synthesis in C. thermocellum. [file DataSheet3.PDF]

Manuscript title: Comparison of transcriptional profiles of *Clostridium thermocellum* grown on cellobiose and pretreated yellow poplar using RNA-Seq

Supplementary Data Sheet 3.

File type: PDF

Contents:

Section 1. Forward (F) and reverse (R) primer sequences for quantitative reverse transcription-PCR (qRT-PCR) analysis of selected genes in *C. thermocellum*.

No. of pages: 1

Section 2. RPKM values of cellulosomal genes based on this study and their protein abundance data retrieved from literature for cellobiose-grown *C. thermocellum* cells at the late stationary phase.

No. of pages: 1

Section 3. List of genes related to cellodextrin transport and phosphorylation, glycolysis, pyruvate catabolism and end-product synthesis in *C. thermocellum*.

No. of pages: 3

Section 1. Forward (F) and reverse (R) primer sequences for quantitative reverse transcription-PCR (qRT-PCR) analysis of selected genes in *C. thermocellum*. FC, fold changes of gene expression in PYP- against cellobiose-grown cells. The presented data for qRT-PCR are means ± SEM of three independent experiments.

Locus ID	Gene name	Primer sequences and the sources	FC	
			mRNA-seq	qRT-PCR
Cthe_1050	RecA (reference gene)	F: GTTGCGGTAAATCTCGATATTGTAAA<br>R: GGCCAATCTTCTGACCGTTG<br>Ref: Stevenson and Weimer (2005)	1.06	1
Cthe_0430	[FeFe] H2ase	F: GGTTGGTGCATGTTGCAGTTAT<br>R: ACAAGCTCCTGGGTTGTCAGA	7.9	5.5 ± 0.3
Cthe_0342	[FeFe] H2ase	F: CCTGTCCCGGTGGTTGTATAA<br>R: TTCGCCCTCTCGCATCTTA	1.3	1.9 ± 0.1
Cthe_3003	[FeFe] H2ase	F: GGTGCGACAAATCCGACAAC<br>R: CGCCGTGAGAGAGGAAAGGT	1.5	2.3 ± 0.2
Cthe_3004	Ferredoxin	F: GGCTGCAATCCGTGTCATT<br>R: ACGCGGCCGCATACTATC	0.6	0.8 ± 0.2
Cthe_3020	ech hydrogenase subunit E	F: GCTGGCTCTCGCCATAGG<br>R: AGGCCTTGGTGTGCTTTCC	0.3	0.3 ± 0.1
Cthe_0394	adhY	F: GACATTGAGGCACGGTCAAA<br>R:: CTCCTGCGTTGGAATTGGTAA<br>Ref: Stevenson and Weimer (2005)	5.3	4.2 ± 0.4
Cthe_2579	adhZ	F: TGACAAAGGAGGAAATTGAGCTT<br>R: CGTGCGTGCATTGGACAA<br>Ref: Stevenson and Weimer (2005)	0.8	1.4 ± 0.1
Cthe_0211	LicB	F: TGCCGGGAGTAACAGGTATGT<br>R: CGGTGGAAACGAGTATTTGCA	19.0	16.4 ± 0.8
Cthe_0412	CelK	F: CAGGTGAAGCATCCCCACAA<br>R: GGGCAGCCTGCGAACTTTAT	8.0	9.2 ± 0.6
Cthe_3077	CipA	F: CAGTATGCTCTTAGTTGTGGCTATGC<br>R: TGATCCAACGGCTGCTGTAA<br>Ref: Stevenson and Weimer (2005)	5.9	5.1 ± 0.3
Cthe_2872	CelG	F: AGCATCCATGTTGGCACTTG<br>R: GACTGCGACGCCTACAAAGC	5.3	4.2 ± 0.4
Cthe_2089	CelS	F: CGCAGAAGGCCGTGCTATA<br>R: CAGAACCTTTACCCTGCTCCTTT<br>Ref: Stevenson and Weimer (2005)	4.5	5.6 ± 0.3

Reference cited in this section: Stevenson, D., and Weimer, P. (2005). *Applied and Environmental Microbiology* 71**,** 4672.

Section 2. RPKM values of cellulosomal genes based on this study and their protein abundance data retrieved from literature for cellobiose-grown *C. thermocellum* cells at the late stationary phase. RPKM, reads per kilobase of exon model per million mapped reads. The definition for emPAI and NSAF and the literature cited were described in the Materials and Methods section.

**LocusID**	**Gene Name**	**RPKM**	**log_2(RPKM)**	**emPAI/CipA (Gold & Martin 2007)**	**NSAF/CipA(Raman et al. 2009)**
Cthe_1400	GH53	11	3.4	0.08	0.11
Cthe_0912	XynY	19	4.3	0.03	0.08
Cthe_0405	CelL	29	4.9	0.15	0.1
Cthe_0745	CelW	40	5.3	0.11	0.36
Cthe_0413	CbhA	49	5.6	0.19	0.77
Cthe_3078	OlpB	59	5.9	0.33	0.11
Cthe_2590	XynD	67	6.1	0.27	0.07
Cthe_0270	ChiA	92	6.5	0.09	0.01
Cthe_0624	CelJ	116	6.9	0.08	0.48
Cthe_1271	GH43, CBM6	116	6.9	0.15	0.04
Cthe_2193	GH5, CBM6	135	7.1	0.29	0.05
Cthe_0032	GH26	159	7.3	0.45	0.07
Cthe_0543	CelF	175	7.5	0.21	0.48
Cthe_2872	CelG	197	7.6	0.25	0.48
Cthe_0412	CelK	202	7.7	0.52	0.77
Cthe_0578	CelR	216	7.8	0.23	0.42
Cthe_2812	CelT	291	8.2	0.12	0.42
Cthe_0433	GH9, CBM3c	306	8.3	0.07	0.23
Cthe_0797	CelE	311	8.3	0.46	0.19
Cthe_0536	CelB	352	8.5	0.38	0.69
Cthe_3077	CipA	363	8.5	1	1
Cthe_1838	XynC	405	8.7	0.61	0.88
Cthe_0269	CelA	1136	10.2	0.56	1.42
Cthe_0821	GH5	1266	10.3	0.53	1.02
Cthe_2972	XynA/U	1357	10.4	0.91	1.31
Cthe_1398	XghA	2827	11.5	0.56	1.25

Section 3. Fold change values for genes related to cellodextrin transport and phosphorylation, glycolysis, pyruvate catabolism and end-product synthesis in *C. thermocellum*. For each gene, fold change (FC) value was calculated by dividing the RPKM of PYP cells by RPKM of cellobiose cells, as listed in Supplementary Data Sheet 1. RPKM, reads per kilobase of exon model per million mapped reads. The table is arranged according to the flux of pathways; within each sub-category, it was arranged in order of FC values. Text in black represents the genes with no significant transcriptional changes between the two types of cells, i.e. 2.0 > FC value > 0.5. Text in red and green represents the genes that found to be significantly up-regulated and down-regulated, respectively, in PYP- against cellobiose-grown cells, based on statistic analysis in Supplementary Data Sheet 2. PYPO, transcript that detected in PYP-grown cells only.

Locus ID	Gene Name	Fold change (PYP/ cellobiose)
**Genes related to cellodextrin transport and phosphorylation**		
Cthe_2125	Cellodextrin (CD) ABC transporter	PYPO
Cthe_1862	Cellodextrin (CD) ABC transporter	10.3
Cthe_0392	Cellodextrin (CD) ABC transporter	8.5
Cthe_0391	Cellodextrin (CD) ABC transporter	7.9
Cthe_0393	Carbohydrate-binding potein A (CbpA)	6.1
Cthe_1019	Cellodextrin (CD) ABC transporter	5.2
Cthe_2126	Cellodextrin (CD) ABC transporter	3.1
Cthe_2128	Carbohydrate-binding potein C (CbpC)	1.2
Cthe_1020	Carbohydrate-binding potein B (CbpB)	1.0
Cthe_2448	Cellodextrin (CD) ABC transporter	0.6
Cthe_2447	Cellodextrin (CD) ABC transporter	0.3
Cthe_2446	Carbohydrate-binding potein D (CbpD)	0.2
Cthe_2989	Cellodextrin phosphorylase (CDP)	1.8
Cthe_1221	Cellobiose phosphorylase (CEP)	3.0
Cthe_0275	Cellobiose phosphorylase (CEP)	1.6
**Genes related to glycolysis, pyruvate catabolism**		
Cthe_1265	Phosphoglucomutase	0.6
Cthe_2938	Glucokinase (GK)	0.9
Cthe_0217	Glucose-6-phosphate isomerase	1.6
Cthe_1261	Phosphofructokinase (PFK)	2.1
Cthe_0347	Phosphofructokinase (PFK)	1.5
Cthe_0349	Fructose-bisphosphate aldolase	0.8
Cthe_0139	Triose phosphate isomerase	0.4

Cthe_0137	Glyceraldehyde-3-phosphate dehydrogenase	0.5
Cthe_0138	3-Phosphoglycerate kinase	0.4
Cthe_1435	Phosphoglycerate mutase (PGM)	2.8
Cthe_0707	Phosphoglycerate mutase (PGM)	2.0
Cthe_1292	Phosphoglycerate mutase (PGM)	1.1
Cthe_0946	Phosphoglycerate mutase (PGM)	1.0
Cthe_0140	Phosphoglycerate mutase (PGM)	0.8
Cthe_2449	Phosphoglycerate mutase (PGM)	0.4
Cthe_0143	Enolase	0.6
Cthe_1253	PEP synthase	2.6
Cthe_1308	PEP synthase	2.0
Cthe_1955	Pyruvate kinase	1.4
Cthe_0505	Pyruvate formate lyase (PFL)	1.4
Cthe_0506	Pyruvate formate lyase activating enzyme (PFL-AE)	2.0
Cthe_1053	l-Lactate dehydrogenase (LDH)	0.9
	Pyruvate:fd oxidoreductase (PFO)	
Cthe_2392	PFO alpha	1.6
Cthe_3120	PFO alpha	1.3
Cthe_2796	PFO alpha	0.3
Cthe_2391	PFO delta	1.7
Cthe_2390	PFO gamma	1.5
**Genes related to end-product synthesis**		
Genes encoding putative hydrogenases and sensory hydrogenases (see Table 4)		
Cthe_1029	Phosphotransacetylase (PTA)	0.2
Alcohol dehydrogenase (ADH)		
Cthe_0423	adhE	7.0
Cthe_0394	adhY	5.3
Cthe_0101	adh	1.5
Cthe_2579	adhZ	0.8
